# Sequential responsive nano-PROTACs for precise intracellular delivery and enhanced degradation efficacy in colorectal cancer therapy

**DOI:** 10.1038/s41392-024-01983-1

**Published:** 2024-10-18

**Authors:** Liuqing Yang, Ye Yang, Jing Zhang, Minghui Li, Long Yang, Xing Wang, Meifang Chen, Hua Zhang, Bing He, Xueqing Wang, Wenbing Dai, Yiguang Wang, Qiang Zhang

**Affiliations:** Beijing Key Laboratory of Molecular Pharmaceutics and New Drug Delivery Systems, State Key Laboratory of Natural and Biomimetic Drugs, School of Pharmaceutical Sciences, Peking University, 100191 Beijing, China

**Keywords:** Drug development, Drug delivery

## Abstract

PROteolysis TArgeting Chimeras (PROTACs) have been considered the next blockbuster therapies. However, due to their inherent limitations, the efficacy of PROTACs is frequently impaired by limited tissue penetration and particularly insufficient cellular internalization into their action sites. Herein, based on the ultra-pH-sensitive and enzyme-sensitive nanotechnology, a type of polymer PROTAC conjugated and pH/cathepsin B sequential responsive nanoparticles (PSRNs) are deliberately designed, following the construction of the PROTAC for Cyclin-dependent kinase 4 and 6 (CDK4/6). Colorectal cancer (CRC) which hardly responds to many treatments even immune checkpoint blockades was selected as the tumor model in this study. As a result, PSRNs were found to maintain nanostructure (40 nm) in circulation and efficiently accumulated in tumors via enhanced permeation and retention effect. Then, they were dissociated into unimers (<10 nm) in response to an acidic tumor microenvironment, facilitating tumor penetration and cellular internalization. Eventually, the CDK4/6 degrading PROTACs were released intracellularly following the cleavage of cathepsin B. Importantly, PSRNs led to the enhanced degradation of target protein in vitro and in vivo. The degradation of CDK4/6 also augmented the efficacy of immune checkpoint blockades, through the upregulation of programmed cell death-ligand 1 (PD-L1) expression in cancer cells and the suppression of regulatory T cells cell proliferation in tumor microenvironment. By combination with α-PD-1, an enhanced anti-tumor outcome is well achieved in CT26 tumor model. Overall, our study verifies the significance of precise intracellular delivery of PROTACs and introduces a promising therapeutic strategy for the targeted combination treatment of CRC.

## Introduction

The advent of PROteolysis TArgeting Chimeras (PROTACs) has revolutionized the field of cancer treatment,^1^ offering an innovative approach to selectively degrade tumor-associated proteins.^2^ PROTACs, which hijack the cellular ubiquitin-proteasome system for protein degradation, usually consist of three components, including small molecules binding to proteins of interest (POIs), E3 ligase ligands and linkers.^3^ Numerous candidates including the prominent ARV-110 and ARV-471 are currently under evaluation in clinical or preclinical studies.^4^ Compared to traditional small molecule inhibitors (SMIs), PROTACs offer notable advantages, such as a catalytic nature, avoiding of drug resistance, and the ability to target proteins previously classified as undruggable.^5^ According to the open-access database PROTAC-DB 2.0, over 3200 PROTACs have been reported to date, targeting 280 POIs.^6^ However, most of them were limited in clinical development due to inherent limitations of PROTACs, such as the undesirable pharmacokinetic properties and insufficient cellular internalization.^7^ Therefore, achieving more effective tumor delivery and robust anti-tumor efficacy remains a significant challenge for PROTACs.

Nanotechnologies have been utilized to address the delivery issues of PROTACs. A well-designed nano-delivery system for tumor-targeted delivery of PROTACs typically involves three stages: 1. accumulation into tumor sites from blood circulation, 2. deep penetration into tumors, 3. internalization into tumor cells and intracellular release to the sites of action. The passive tumor accumulation and enhanced cellular uptake of PROTACs could be achieved via enhanced permeation and retention (EPR) effect and ligands modification of nanoparticles, respectively.^8,9^ Besides, many efforts have been made to further improve the circulatory stability and tumor penetration of PROTACs, as well as to introduce other therapeutic modalities for combination therapies. For example, Yu et al. developed a combined delivery system (POLY-PROTAC), which significantly enhanced tumor penetration of PROTACs through biological orthogonal reaction.^10^ Pu et al. designed the SPNpro, which combined PROTACs with photothermal therapies and effectively inhibited the development of tumors.^11–13^ It’s noteworthy that PROTACs mainly function in the tumor cytoplasm.^14^ If the drug is released at the extracellular space, it’s still hard to overcome the inherent limitation of PROTACs. To maximize PROTAC exposure at the site of action, it seems necessary to design a reasonable delivery system to achieve prolonged circulation, tumor penetration, cellular internalization and intracellular release of PROTACs simultaneously. However, there are currently no reports meeting all these requirements.

Previously, we developed an ultra-pH-sensitive (UPS) nanotechnology, demonstrating its versatility in biomedical applications such as image-guided surgical resection and tumor-specific delivery of drugs.^15,16^ These UPS particles maintained their structural integrity during circulation and immediately dissociated into smaller unimers in response to subtle changes of pH (∆pH <0.25).^17^ This characteristic has been proven to be favorable for drug delivery system in terms of prolonged circulation, tumor targeting and penetration. In solid tumors, the high glycolytic activity of cancer cells (known as Warburg effect) and poor vascular perfusion lead to an overaccumulation of acidic metabolic products, creating an acidic tumor microenvironment (TME, pH 6.5–7.0).^18^ The acidic conditions within TME not only impact the progression of tumors,^19^ but are harnessed to design the pH-responsive PROTAC delivery system. Additionally, we utilized a tetrapeptide Gly-Phe-Leu-Gly (GFLG), which could be cleaved by cathepsin B highly expressed in lysosomes, as a responsive linker to connect PROTACs.^20^ Such a design facilitates the intracellular release of PROTACs from the internalized nanoparticles in response to cathepsin B, further enhancing the exposure of PROTACs at their action sites.

Colorectal cancer (CRC) is the third most common malignancy.^21^ For most CRC patients, many treatments including immune checkpoint blockades (ICBs) fail to achieve significant efficacy, probably owing to low infiltration of immune cells, increase of immunosuppressive cells like regulatory T (Treg) cells and low abundance of programmed cell death-ligand 1 (PD-L1) in tumors.^22^ Recent studies related to its combined therapy have demonstrated the possibility of improving anti-tumor activity.^23^ Cell-cycle control is frequently dysregulated in malignant tumors.^24^ Cyclin-dependent kinase 4 and 6 (CDK4/6) play an important role in the transition from G1 to S phase.^25^ SMIs of CDK4/6 such as palbociclib have been approved for the metastatic hormone receptor-positive breast cancer and are currently undergoing clinical trials for CRC.^26,27^ However, an acquired resistance and a compensatory increase in CDK4/6 expression have been observed with these inhibitors.^28,29^ Thus, replacing SMIs with CDK4/6 targeted PROTACs might be a solution to avoid drug-resistance.^30^ In this study, we synthesized a CDK4/6-targeting degrader chimera as a model PROTAC for CRC therapy.^31^ We then designed and prepared PROTAC conjugated and pH/cathepsin B sequential responsive nanoparticles (PSRNs). PSRNs were assembled by diblock copolymers (PEG-*b*-P(EPA-*r*-PROTAC)) in which the PROTAC coupled to the copolymer via a cathepsin B responsive sequence (GFLG). After intravenous administration, PSRNs maintained their nano-structure and exhibited prolonged blood circulation. Upon accumulated at tumor sites, PSRNs dissociated into unimers at the acidic TME. After internalization by tumor cells, PROTAC were released intracellularly by cleavage of cathepsin B in lysosomes. Herein, PSRNs had overcome multiple biological barriers and delivered PROTAC to its active sites. On the one hand, inhibition of CDK4/6 is expected to sensitize CRC to immune checkpoint blockades (ICBs) through upregulation of PD-L1 level^32^ and reduction of immunosuppressive Treg population.^33,34^ On the other hand, inhibition of CDK4/6 realized the strong anti-tumor efficacy. In this study, considering PSRNs achieved both anti-tumor and immune-effects, we investigated the therapeutic potential of PSRNs in combination with ICBs in CT26 tumor models.

## Results

### Preparation and characterization of PSRNs

**Fig. 1:**
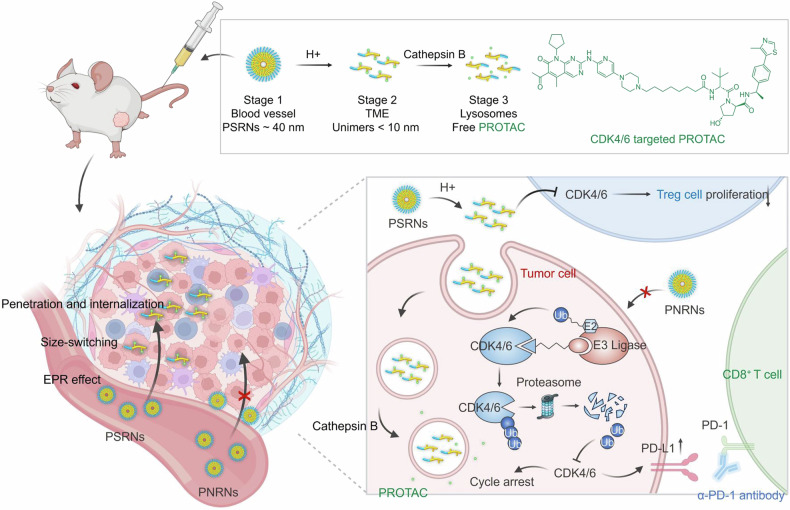
The structure of CDK4/6-targeted PROTAC. The schematic illustration of the sequential responsive process of PSRNs after intravenous administration. The proposed behavior of protein-degradation and immunoregulation post PSRNs and ICBs combination therapy in the tumor-bearing mice. Created with *BioRender.com*

**Fig. 2:**
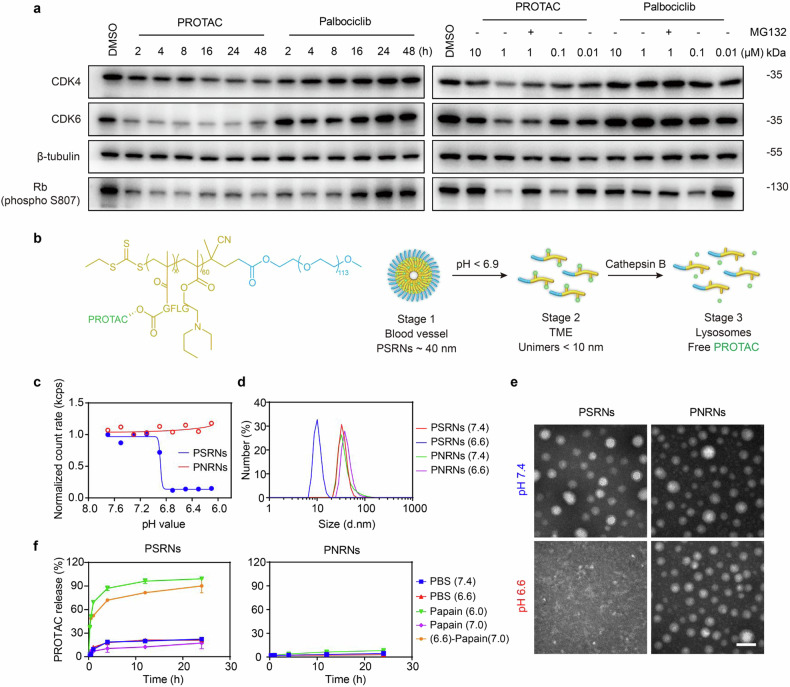
Preparation and characterization of the CDK4/6 degrading PSRNs. Time-dependent experiment (**a**, left) of CDK4/6 degradation and Rb (phospho S807) downregulation in CT26 cells (treated at 1.0 µM for the indicated time). PROTAC induces CDK4/6 degradation in a concentration-dependent manner (**a**, right) after incubation of 16 h (MG132 used as proteasome inhibitor at 0.5 µM). β-tubulin was used as loading control. **b** Schematic illustration of the sequential pH/cathepsin B responsiveness of PSRNs. **c** Count rates of PSRNs and PNRNs at different pH (count rates was normalized to that determined at pH 7.4, *n* = 1). Dynamic light scattering (DLS) profiles (**d**) and TEM images (**e**) of PSRNs and PNRNs at pH 7.4 or 6.6, scale bar = 50 nm. **f** In vitro PROTAC release from PSRNs and PNRNs in different medium. (6.6)-papain (7.0) indicates the pH of release medium was adjusted to 6.6 in advance, then back to 7.0 with addition of papain. PROTAC release after incubation of 24 h with excess papain (80 μM) was regarded as 100%. Mean ± SD (*n* = 3)

We successfully synthesized a Von Hippel-Lindau (VHL) based CDK4/6-targeting PROTAC (Fig. 1 and Supplementary Fig. 1), which has the capacity of CDK4/6 protein degradation and phosphorylated retinoblastoma (Rb (phospho S807)) downregulation in mice CRC CT26 cells (Fig. 2a) and human breast cancer MDA-MB-231 cells (Supplementary Fig. 2) in a concentration- and time-dependent manner. Conversely, cells treated with the small molecular inhibitor (palbociclib) exhibited a compensatory increase in CDK4/6 levels instead of degradation. The addition of MG132 (used as a proteasome inhibitor) reversed the capacity of PROTAC-mediated protein degradation, indicating that the degradation of CDK4/6 by PROTAC occurred through a ubiquitin-proteasome-dependent pathway (Fig. 2a and Supplementary Fig. 2).

Next, to synthesize the pH/cathepsin B dual-responsive polymer-PROTAC conjugate PEG-*b*-P(EPA-*r*-PROTAC), pH-sensitive monomer EPA-MA and cathepsin-B-responsive monomer MA-GFLG-PROTAC were firstly synthesized. Their chemical structures and purity were confirmed by mass spectrometry, proton nuclear magnetic resonance spectra (^1^H-NMR) and high performance liquid chromatography (HPLC) (Supplementary Figs. 3, 4). Then, PEG-*b*-P(EPA-*r*-PROTAC) was synthesized by reversible addition-fragmentation chain transfer (RAFT) polymerization method (Supplementary Fig. 5a). The coupling of PROTAC to polymer was verified by ultraviolet–visible (UV-Vis) scanning (Supplementary Fig. 5b). Gel permeation chromatography was used to analyze the molecular weight of the polymer (M*w*: 22.7 kDa) (Supplementary Fig. 5c and Supplementary Table 1), and ^1^H-NMR was used to verify the structure of the polymer (PROTAC of 7.2%) (Supplementary Fig. 5d).

A pH-nonresponsive polymer–PROTAC conjugate PEG-*b*-P(EH-*r*-PROTAC) (M*w*: 26.5 kDa, PROTAC of 7.3%) (Supplementary Fig. 6), a pH-responsive polymer PEG-*b*-P(EPA) and a pH-nonresponsive polymer PEG-*b*-P(EH) were also synthesized for comparison (Supplementary Fig. 7). Then, PSRNs and PNRNs (PROTAC conjugated and pH-nonresponsive nanoparticles) were self-assembled by PEG-*b*-P(EH-*r*-PROTAC) and PEG-*b*-P(EPA-*r*-PROTAC), respectively.

PSRNs and PNRNs maintained Z-average diameter of 41.5 and 53.8 nm at pH 7.4, with polydispersity index (PdI) of 0.270 and 0.228, respectively (Supplementary Fig. 8a). For investigating the pH responsiveness of PSRNs in acidic environment, we adjusted the pH of dispersion medium of nanoparticles. As shown in Fig. 2c, for PSRNs, count rates keep constant at pH ≥7.0 (physiological pH), whereas, with pH lower than 6.9, there was a significant decrease of count rates, as a result of protonation of the EPA blocks and dissociation of the nanoparticles. The TME of CRC was reported a pH of 6.8.^35^ Transition pH of 6.9 allows PSRNs for the precise response to the acidic TME. At acidic pH (6.6), PSRNs completely dissociated into amorphous unimers with sizes of about 8.9 nm, while PNRNs maintained the same size instead (Fig. 2d). Transmission electron microscopy (TEM) images also exhibited an evident pH-induced dissociation of PSRNs but not of PNRNs (Fig. 2e). Furthermore, an increase of zeta-potential of PSRNs was observed at pH 6.6 (Supplementary Fig. 8b). The increase in zeta-potential was advantageous for facilitating the cellular uptake of PSRNs.

Finally, to evaluate pH/enzyme sequential responsive PROTAC release from PSRNs in vitro, the samples were incubated with different media (Fig. 2f). The results showed that PROTAC was almost not released in PBS of pH 6.6 and pH 7.4 without papain. For the media of pH 6.0 with papain, it was observed that there was an obvious and rapid PROTAC release over 24 h. Thus, PROTAC release from PSRNs was enzyme-responsive. The most suitable pH for papain is 6-7. It was found that PROTAC would not be released in neutral environment (pH 7.0) even though the release medium contained the papain. However, PROTAC could be released if we adjusted pH of medium to 6.6 in advance, then back to 7.0 with addition of enzymes. The results evidently indicated that the PROTAC release from PSRNs not only need both the slightly acidic environment and enzyme degradation, but the dissociation triggered by weak acidic pH should be prior to the cleavage of conjugate by enzyme. As shown in Fig. 2f, PROTAC was released rapidly in papain at pH 6.0, with over 80% of drug was released from PSRNs within a 4 h period. In contrast, in all media, the release of PROTAC from PNRNs remained below 10% after 24 h incubation. It might be that PNRNs maintained their nanostructure which hindered the enzymatic cleavage ability of papain. In short, we designed, prepared and characterized the PSRNs. The elaborately designed stimuli-responsive dissociation and unique release behavior of PSRNs provided basis for their further application.

### PSRNs improve CDK4/6 degradation efficacy in vitro

Next, the action procedure of the PSRNs was evaluated in vitro. The biosafety of non-PROTAC nanoparticles PEG-*b*-P(EPA) and PEG-*b*-P(EH) were firstly evaluated in CT26 tumor cells under medium of pH 7.4 and pH 6.6. Both PEG-*b*-P(EPA) and PEG-*b*-P(EH) exhibited limited cytotoxicity at a concentration up to 1 mg/mL (Supplementary Fig. 9).

**Fig. 3:**
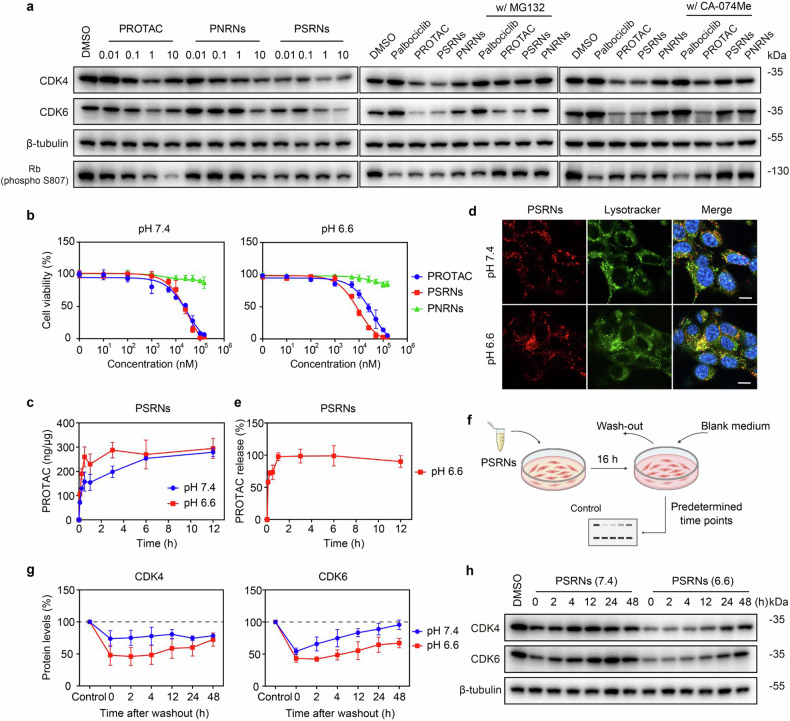
Improvement of CDK4/6 degradation efficacy in vitro. **a** Western blot assay of CDK4/6 degradation in CT26 cells. (Left) Expression of CDK4/6 in CT26 cells treated with different concentrations (μM) of PROTAC formulations after incubation of 16 h. (Middle) Expression of CDK4/6 in CT26 cells treated with (w/) or without (w/o) MG132 (PROTAC concentrations of 1.0 μM and MG132 concentration of 0.5 μM). The w/ and w/o indicate cells were treated with both MG132 and PROTAC formulations or PROTAC formulations alone for 16 h. (Right) Expression of CDK4/6 in CT26 cells treated w/ or w/o CA-074Me preincubation (PROTAC concentrations of 1.0 μM and CA-074Me concentration of 20 μM). The w/ indicates cells were pre-treated with CA-074Me for 4 h and then incubated with PROTAC formulations for 12 h. The w/o indicates cells were incubated with PROTAC formulations alone for 12 h. β-tubulin was used as loading control. **b** Cell viability of CT26 cells at pH 7.4 or 6.6 after 72 h incubation with PROTAC formulations. Mean ± SD (*n* = 4). Uptake of PSRNs (**c**) and relative intracellular release of PROTAC (**e**) in CT26 cells at pH 7.4 or 6.6. Mean ± SD (*n* = 3). **d** Confocal laser scanning microscope (CLSM) images of intracellular colocalization of PSRNs (red) with lysosomes (green, stained with lysosome tracker) in CT26 cells at pH 7.4 or 6.6. Nuclei were stained with Hoechst 33342 (blue). Scale bar = 10 µm. **f** Diagrammatic illustration of wash-out assay in **h** and **g**. Western blot (**h**) and quantification (**g**) of change of CDK4/6 expression in CT26 cells in wash-out assay. Cells were incubated for indicated duration after drug-containing medium replaced by blank medium (PROTAC concentrations of 1 μM). β-tubulin was used as loading control. Mean ± SD (*n* = 3)

Effective cellular internalization and drug release were prerequisites for efficient protein degradation by PSRNs. Firstly, we investigated the cellular uptake of PSRNs in vitro. For visualization of preparations, we synthesized fluorescently labeled PEG-*b*-P(EPA)-Cy5 and PEG-*b*-P(EH)-Cy5 (Supplementary Fig. 10). Our previous study^15^ showed that, “always on” PSRNs (Supplementary Fig. 10c) could be prepared by adding 20% fluorescently labeled polymer into formulations, and used for semi-quantitative analysis of delivery behavior in vitro and in vivo. The exposure of positive charges and reduction of size were beneficial for cellular internalization. In CT26 cells, the uptake of PSRNs at all time points was obviously higher at pH 6.6 than that at pH 7.4 (Supplementary Fig. 11a). In contrast, PNRNs showed poor cellular uptake (Supplementary Fig. 11b), with no significant changes at different pH values. These findings were further confirmed by liquid chromatography-mass spectrometry (LC-MS). Moreover, the internalization rate of PSRNs within 1 h incubation at pH 6.6 was 1.5-fold higher than that at pH 7.4 (Fig. 3c). The endocytosis inhibition assay showed that the internalization of PSRNs was mainly through clathrin- and caveolin-mediated endocytosis, requiring energy expenditure (Supplementary Fig. 11c).

Based on current knowledge, PSRNs are expected to be trapped into lysosomes following cellular internalization. As shown in Fig. 3d, we observed that red punctate PSRNs signals almost completely colocalized with green lysosomes signals in CT26 cells after incubation of 2 h. Given the high abundance of cathepsin B in the lysosomes, a cathepsin B responsive conjugation design was integrated in the UPS system to enhance the efficient intracellular release of PROTAC. In previous section we have confirmed the enzyme-responsive degradation of PSRNs for the release of PROTAC in vitro. Here, we proceeded to evaluate the PROTAC release from PSRNs following cellular internalization by LC-MS analysis. It was observed that over 90% of PROTAC could be rapidly released within 1 h in CT26 cells at pH 6.6 (Fig. 3e). Effective cellular internalization and intracellular release of PROTAC were crucial for protein degradation efficacy of PROTAC.

Then, we investigated CDK4/6 protein degradation efficacy of PSRNs in vitro. As shown in Fig. 3a and Supplementary Fig. 12a–c, both free PROTAC and PSRNs demonstrated efficient protein degradation capability in a variety of tumor cells. By contrast, PNRNs exerted limited protein degradation efficacy even at a high concentration, due to poor release of PROTAC and poor cellular internalization. Similar to the mechanism of free PROTAC, co-incubation with MG132 (proteasome inhibitor) could reverse the protein degradation effects of PSRNs in a concentration-dependent manner (Fig. 3a and Supplementary Fig. 12d), indicating the degradation of CDK4/6 by PSRNs occurred through a ubiquitin-proteasome-dependent pathway. Moreover, the reduction of CDK4/6 degradation in PSRNs-treated CT26 cells pre-incubation with CA-074Me (cathepsin B inhibitor) demonstrated that PROTAC was released from PSRNs mainly via cleavage of cathepsin B.

With curiosity, we evaluated the change of CDK4/6 expression in CT26 cells after washing-out of drug-containing medium in vitro (Fig. 3f). The results showed that PSRNs, instead of free PROTAC and PNRNs, exerted enhanced CDK4/6 degradation capability in acidic environment (Fig. 3g, h and Supplementary Fig. 13). And it was demonstrated that the recovery rate of protein expression in cells treated with PSRNs was significantly lower than that in cells treated with both free PROTAC and PNRNs. According to the semi-quantitative results by ImageJ, the CDK4/6 degradation percentage of PSRNs at pH 6.6 was 2.1- and 2.3-fold higher than that at pH 7.4, respectively, and was 2.4- and 1.6-fold higher than that of free drugs in the same medium (Supplementary Table 2).

Finally, the cytotoxicity of PROTAC preparations on tumor cells was evaluated. As expected, the inhibition of CDK4/6 in CT26 cells induced G1 cell cycle arrest (Supplementary Fig. 14). As shown in Fig. 3b and Supplementary Fig. 15, both free PROTAC and PROTAC-conjugated preparations exhibited dose-dependent cytotoxicity. Similar to results of CDK4/6 degradation efficacy, PNRNs demonstrated poor cytotoxicity. By contrast, PSRNs exerted excellent cytotoxicity, especially at pH 6.6, similar to or beyond that of free PROTAC. The IC_50_ value of PSRNs in CT26 cells at pH 6.6 was 3.1-fold lower than that of free PROTAC at the same medium (Supplementary Table 3). Meanwhile, it was revealed that both free PROTAC and preparations inhibited proliferation only at high PROTAC concentrations in mouse fibroblast L-929 and human umbilical vein endothelial (HUVEC) cells (Supplementary Fig. 15), due to different expression levels of CDK4/6 in tumor cells and nontumor cells (Supplementary Fig. 16).

Altogether, size-switchable and appropriate responsive characteristics make PSRNs easier be internalized by tumor cells and effectively release drugs intracellularly. Enhanced PROTAC intracellular release leaded to improvement of PROTAC-mediated CDK4/6 protein degradation and cytotoxicity in vitro.

### PSRNs display enhanced penetration and tumor targeting capability in vitro and in vivo

**Fig. 4:**
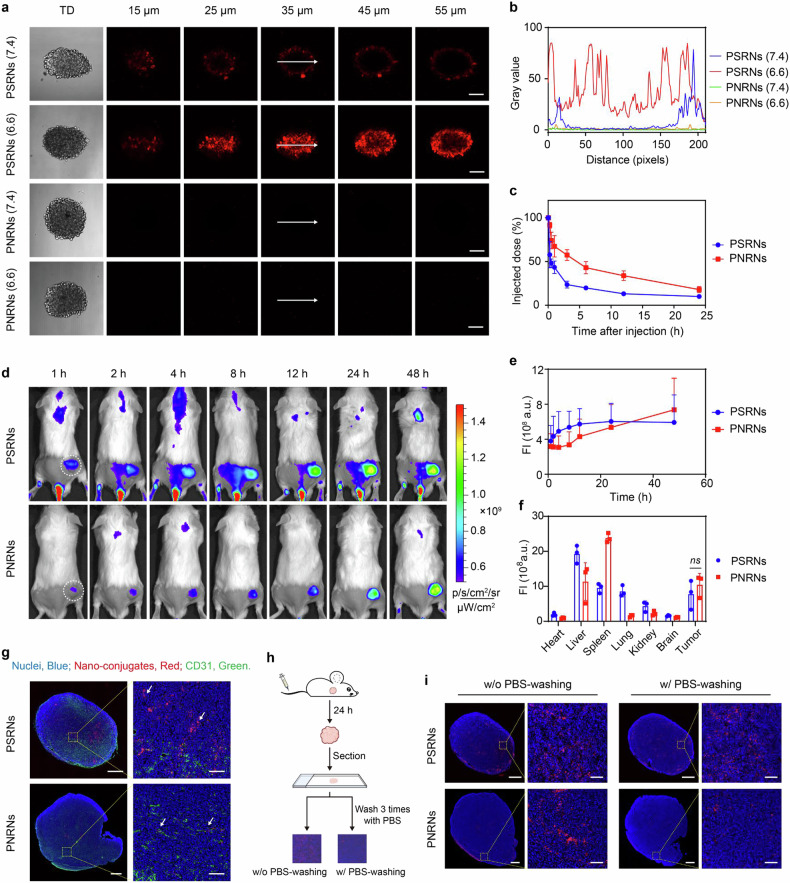
Enhanced tumor-penetration and accumulation in vitro and in vivo. **a** CLSM Z-stack scanning of CT26 multicellular tumor spheroids (medium permeability) after incubation with PSRNs and PNRNs at pH 7.4 and 6.6 for 8 h. The surface of the spheroids was defined as 0 µm. Scale bar = 100 μm. **b** Cy5 fluorescence intensity of CT26 tumor spheroids in **a** along the white arrow. Analyzed by ImageJ. **c** Pharmacokinetics profiles after intravenous administration of PSRNs and PNRNs in health BALB/c mice. Mean ± SD (*n* = 5). **d** In vivo fluorescence images of CT26-tumor-bearing BALB/c mice at different time points after the intravenous injection of PSRNs and PNRNs. **e** Fluorescence intensity (FI) of PSRNs and PNRNs distribution at tumor sites. Mean ± SD (*n* = 3). **f** Ex vivo fluorescence intensity of tumors and major organs in CT26-tumor-bearing BALB/c mice at 48 h after intravenous injection of PSRNs and PNRNs. Mean ± SD (*n* = 3). **g** Whole-scanning fluorescence images of tumor sections from CT26-tumor bearing mice at 24 h post-injection revealed the penetration from blood vessels of PSRNs and PNRNs. Left panel scale bar = 800 μm, right panel scale bar = 100 μm. **h** Diagrammatic illustration of assay in **i**. **i** Whole-scanning fluorescence images of tumor sections from CT26-tumor-bearing BALB/c mice at 24 h post-injection of PSRNs and PNRNs, sections were treated w/o or w/ PBS-washing before capture. Left panel scale bar = 800 μm, right panel scale bar = 100 μm

To achieve effective anti-tumor efficacy, nanoparticles must accumulate and penetrate at tumor tissues efficiently.^36^ Here, we continued to study its penetration and targeting capability in vitro and in vivo. It is known that nanoparticles accumulated at tumor sites through the EPR effect. Based on this, size switching in the acidic TME helps PSRNs improve tumor penetration. To test this hypothesis, we utilized 3D multicellular tumor spheroids as an in vitro model to evaluate the penetration efficacy. Specifically, human breast cancer MCF-7, mouse CRC CT26, and pancreatic cancer PANC-1 tumor spheroids were used as high, medium and low penetration 3D tumor models, respectively. Tumor spheroids were incubated with Cy5-labeled PSRNs for 8 h at pH 7.4 and 6.6, and then captured by CLSM Z-stack scanning. It was difficult to detect fluorescence signal from PNRNs at both pH conditions. Very weak fluorescence was detected only in MCF-7 tumor spheres, which was mostly located at the edges of tumor spheres. In contrast, fluorescence intensity (FI) of samples treated with PSRNs were obviously higher than that treated with PNRNs, which was owed to increase of cellular uptake. More importantly, at pH 6.6, with dissociation into smaller particles, PSRNs’ fluorescence signal reached 45 μm into tumor spheres (Fig. 4a, b and Supplementary Fig. 17), demonstrating a high penetration capability for PSRNs.

Next, we studied the pharmacokinetics of PSRNs and PNRNs in health BALB/c mice (Fig. 4c). Their elimination half-lives were 12.5 ± 7.2 and 13.7 ± 1.0 h, respectively (Supplementary Table 4). PNRNs with strong hydrophobic cores displayed prolonged circulation time, compared to PSRNs. Then, the tissue distribution of PSRNs and PNRNs were investigated by in vivo imaging. As shown in Fig. 4d, e, both the PSRNs and PNRNs quickly accumulated at tumor site after injection, and the amounts increased considerably within 48 h. Obviously, the persistent tumoral accumulation was attributed to their prolonged blood circulation. The major organs and tumor tissues were harvested for ex vivo imaging (Fig. 4f and Supplementary Fig. 18). As shown in Fig. 4f, the fluorescence intensity of tumors at 48 h after intravenous injection of PSRNs and PNRNs were 10.2 and 12.7 times higher than that of surrounding muscles, respectively.

We further examined the intratumor penetration of preparations by frozen section. Although there was a good accumulation for PNRNs at tumor site, very weak fluorescence was observed from sections and was mostly located in vessels and failed to penetrate into tumor spaces (Fig. 4g). In contrast, PSRNs diffused into deep regions from the tumor vessels. We further observed that fluorescence intensity of tumors treated with PNRNs significantly decreased after washing with PBS (Fig. 4h, i). Based on the above results, we could conclude that although PNRNs could accumulated in the tumor site through EPR effect, they might not be internalized by tumor cells. In contrast, washing with PBS had no significant effect on flurescence signal of tumor section from PSRNs group (Fig. 4i).

Altogether, PROTAC-conjugated nanoparticles displayed efficient tumor-specific accumulation via EPR effect. However, only PSRNs with size-switchable capability could further penetrate into the deep tumor and are internalized by the tumor cells in vivo. PSRNs we designed overcame multiple barriers for delivering PROTAC deeply into tumor tissues, were available for stronger protein degradation efficiency and anti-tumor efficacy.

### PSRNs enhanced the susceptibility of CT26 tumors to ICBs

**Fig. 5:**
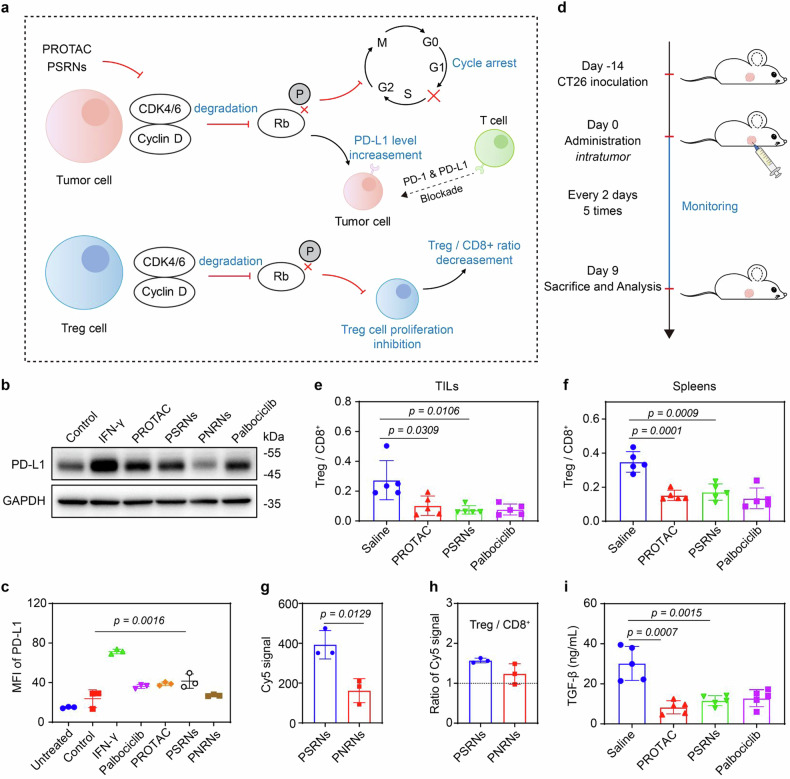
PSRNs enhanced the susceptibility of CT26 tumors to ICBs. **a** Schematic illustration of improvement of immunosuppressive microenvironment with treatment of CDK4/6 degradation. Rb, short for retinoblastoma protein. P, short for phosphorylated. **b** Western blot assay of expression of PD-L1 in CT26 cells with post-incubation of 48 h. PROTAC and palbociclib concentrations of 1 μM, interferon-γ (IFN-γ, 2 ng/mL) used as positive control. GAPDH was used as loading control. **c** Mean fluorescence intensity (MFI) of PD-L1 expression in CT26 cells with post-incubation of 48 h analyzed by flow cytometry. PROTAC and palbociclib concentrations of 1 μM, IFN-γ (2 ng/mL) used as positive control. Mean ± SD (*n* = 3). **d** Therapeutic schedule (related to **e-f** and **i**) for immune response analysis in CT26 tumor-bearing BALB/c mice (*intratumoral* administration, PROTAC of 2 mg/kg). Treg cells (CD4^+^CD25^+^FoxP3^+^)/CD8^+^ T cells ratios analyzed by flow cytometry of tumors (**e**) and spleens (**f**) in CT26 tumor-bearing BALB/c mice. Mean ± SD (*n* = 5). TILs mean Tumor infiltrating lymphocytes. Fluorescence intensity in Treg cells (**g**) and ratio of fluorescence intensity of Treg / CD8^+^ T cells (**h**) in CT26 tumors at 24 h post-injection analyzed by flow cytometry. Mean ± SD (*n* = 3). **i** Levels of TGF-β in plasma of mice treated as therapeutic schedule in **d** by ELISA assay. Mean ± SD (*n* = 5)

Most CRC patients are not benefited from immunotherapy, including ICBs, due to the increase of immunosuppressive cells (such as Treg cells) and the low abundance of PD-L1.^37^ It was found that, inhibition of CDK4/6 could enhance the susceptibility of CT26 tumors to ICBs (Fig. 5a).

Firstly, we evaluated PD-L1 expression level in CT26 tumor cells after incubation with PSRNs. Results from both western blot assay and flow cytometry revealed that degradation of CDK4/6 increased PD-L1 protein levels in CT26 cells (Fig. 5b, c). A high proportion of Treg cells in tumors predicted a poor clinical prognosis.^38,39^ Then, to investigate inhibitation of Treg cells after CDK4/6 PROTAC treatment in vivo, CT26 tumor bearing mice were employed. Particles with smaller size were easier taken up by T cells.^40,41^ Compared to PNRNs, PSRNs could be taken up easier by Treg cells as a result of dissociation in TME (Fig. 5g). Moreover, uptake of PSRNs by Treg cells were 1.6-fold higher than CD8^+^ T cells (Fig. 5h). After 5 times administration with CDK4/6 targeted PROTAC intratumorally (Fig. 5d and Supplementary Fig. 19), we found that, both in tumors and spleens, Treg /CD8^+^ T cells ratios were significantly decreased (Fig. 5e, f), demonstrating an efficient regulation of immunosuppresive TME. The results were further surpported by decrease of transforming growth factor β (TGF-β) level in serum (Fig. 5i).

All these data supported that PSRNs could enhance the susceptibility of CT26 tumors to ICBs. All provided a molecular raionale for combining CDK4/6 PROTAC treatment with immunotherapy by α-PD-1.

### In vivo anti-tumor efficacy of PROTACs based PSRNs combined with immunotherapy

**Fig. 6:**
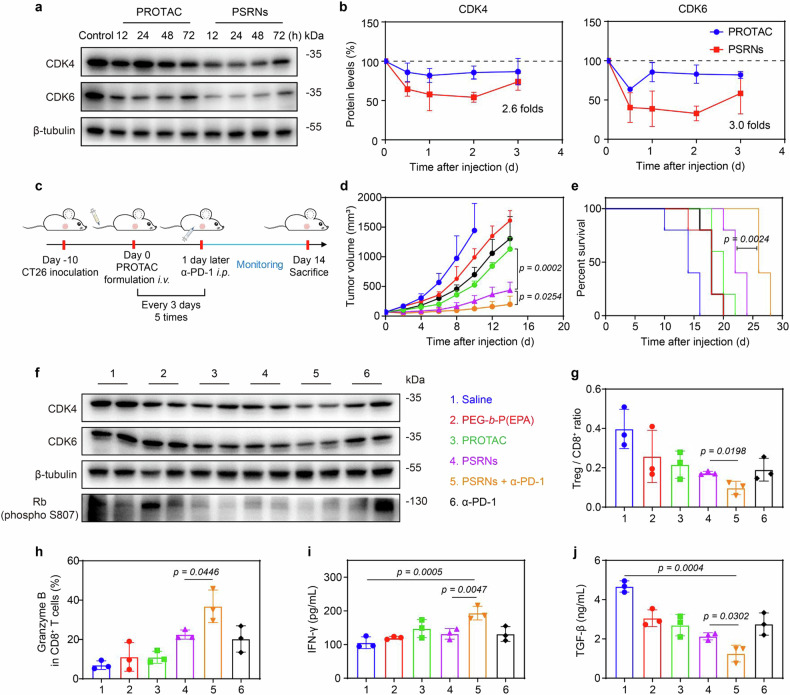
In vivo anti-tumor efficacy of PSRNs combined with immunotherapy. Western blot assay (**a**) and quantification (**b**) of change of CDK4/6 expression in CT26 tumors at different time points after single administration (PROTAC dosage of 5 mg/kg, *i.v*.). PSRNs exerted 2.6- and 3.0-fold higher CDK4/6 degradation efficacy than that of free PROTAC, respectively. β-tubulin was used as loading control. Mean ± SD (*n* = 3). **c**. Experiment schedule (related to **d**) for anti-tumor study in CT26 tumor-bearing BALB/c mice (PROTAC of 5 mg/kg, α-PD-1 of 50 μg/each, *n* = 5). **d** Averaged tumor growth curves of CT26-tumor bearing BALB/c mice after treatment as schedule in **c**. Due to the observation of tumor volume exceeding 2000 mm^3^, samples were collected in advance. Mean ± SD (*n* = 5). **e** Survival curves of CT26-tumor bearing BALB/c mice after treatment as schedule in Supplementary Fig. 23a. Mean ± SD (*n* = 5). **f** Western blot assay of CDK4/6 degradation in CT26 tumors. Treg cells/CD8^+^ T cells ratios (**g**) and percents of granzyme B^+^ T cell populations (**h**) in tumor tissues quantified by flow cytometry analysis. Mean ± SD (*n* = 3). Levels of IFN-γ (**i**) and TGF-β (**j**) in tumor homogenate quantified by ELISA assay. Mean ± SD (*n* = 3)

After demonstrating the protein degradation efficacy and cytotoxicity of PSRNs in vitro, we proceeded to examine the impact of the pH/cathepsin sequential responsive delivery strategy on in vivo degradation and anti-tumor efficacy. Firstly, the protein degradation efficacy post single *i.v*. administration was investigated in CT26 tumor-bearing mice. As shown in Fig. 6a, b, PSRNs exerted 2.6- and 3.0-fold higher CDK4/6 degradation effects compared to free PROTAC, respectively (Supplementary Table 5). Similar results were supported by CLSM images (Supplementary Fig. 20). Notably, PSRNs exerted prolonged CDK4/6 degradation efficacy in vivo.

Then, we evaluated the anti-tumor efficacy of PSRNs in BALB/c mice bearing a CT26 tumor xenograft (Fig. 6c). The utilization of a pH/cathepsin B reponsive delivery system significantly improved the anti-tumor efficacy of PROTAC, compared with free PROTAC group (Fig. 6d and Supplementary Fig. 21). Western blot assay revealed a significant decrease in CDK4/6 expression in tumors (Fig. 6f). Hematoxylin and eosin (H&E) staining demonstrated an increase of tumor necrosis after PSRNs treatment, indicative of improved anti-tumor efficacy (Supplementary Fig. 22a). Recently, the combination of ICBs and other therapies has been confirmed in many tumor models.^42^ It was proved that treatment with CDK4/6-targeted PROTAC could enhance the susceptibility of CT26 tumors to ICBs. Thus, to enhance anti-tumor efficacy, diversify therapeutic approaches of PROTAC, we combined PSRNs with ICBs. The combination treatment further enhanced the anti-tumor efficacy, extending the median survival time (MST) from 22 to 26 days (Fig. 6e, Supplementary Fig. 23 and Supplementary Table 6). No noticeable tissue damage was ovserved in marjor organs upon H&E staining, suggesting the safety of therapeutical strategy (Supplementary Fig. 22b). Flow cytometry analysis after treatment revealed a decrease in Treg/CD8^+^ ratio and an increase in GranB^+^ CD8^+^ T cell population in the combination therapy group, revealing the alleviation of immunosuppressive TME and an increase of tumor infiltrating lymphocytes (Fig. 6g, h). It was further surpported by a decrease of TGF-β and an increase of IFN-γ level in serum and tumor homogenate (Fig. 6i, j and Supplementary Fig. 21e, f).

Altogether, PSRNs not only enhance the penetration and uptake of PROTAC in tumors, thus enhancing anti-tumor efficacy, but also be additional with immunotherapy to further broaden the treatment methods of PROTAC and improve its anti-tumor efficacy.

## Discussion and conclusion

Recently, PROTACs have shown promising potential in cancer therapy.^43^ However, their clinical translation of PROTACs is hindered by limited cellular internalization and undesirable tissue specificity. VHL-based PROTACs particularly owing to their high molecular weight with many hydrogen bond donors and acceptors, exhibit a tendency of diminished permeability and limited cellular internalization, which could hinder their therapeutic potential.^44^ Therefore, it is necessary to design a reasonable delivery system to overcome unfavorable physicochemical properties of the PROTACs and achieve strong anti-tumor efficacy. In this study, we developed PROTAC conjugated pH/cathepsin B sequential responsive nanoparticles (PSRNs) for PROTACs therapy, which demonstrated significantly enhanced PROTAC intracellular release (Fig. 3) and improved anti-efficacy in CT26-tumor bearing mice (Fig. 6).

PROTACs hijack the cellular ubiquitin-proteasome system for protein degradation, their primary action occurs in the cytoplasm.^14^ Therefore, in the design of delivery systems, it is important to systematically overcome physiological barriers, but put more focus on achieving precise intracellular drug release. PSRNs we designed effectively surmount multiple barriers by sequentially responding to the acidic TME and intracellular enzyme, facilitating release of PROTACs within cells. The results, including in vitro drug release (Fig. 2f), intracellular drug content (Fig. 3e), and in vivo studies in animal models (Fig. 4), all demonstrated the existence and significance of this sequential response. PSRNs maintained their nanostructure (40 nm) in neutral circulation. With presence of PEG, the circulation time was prolonged. Upon reaching the tumor site, PSRNs rapidly dissolved into smaller unimers (<10 nm) in response to acidic TME, firstly. Size-switchable nanoparticles penetrate into the tumors and internalized by tumor cells. Within cells, PSRNs were cleaved by cathepsin B in the lysosomes, facilitating the precise intracellular delivery of PROTACs. If the sequence of response was changed or simultaneous, it might be extracellular release of PROTACs, still cannot solve the problem of PROTACs endocytosis. Therefore, the sequential response of PSRNs is important.

PSRNs significantly enhanced PROTAC intracellular release. Enhanced exposure of PROTACs in action site enhanced protein degradation efficacy. It was found that if PROTACs was wash-out, the POIs that had been degraded would rapidly recover. Limited degradation time leaded to increased frequency of administration in clinics. It might due to the insufficient intracellular PROTACs.^45^ The results in vitro (Fig. 3h) and in vivo (Fig. 6a) demonstrated PSRNs enhanced protein degradation efficacy and extended protein recovery time, which might be beneficial for reducing the frequency of administration.

The synthesized CDK4/6-targeting PROTAC in our study exhibited degradation effects on various tumors (Supplementary Fig. 12). As to CRC, most patients show high expression levels of CDK4/6, and with low mutation rate of key protein in downstream.^46–48^ Therefore, CDK4/6 are important therapeutic targets for CRC therapy. Combination therapeutic strategies of CDK4/6 degraders are explored, recently.^49^ On this basis, we took advantage of fact that degradation of CDK4/6 could enhance the sensitivity of ICBs, including upregulation of PD-L1 expression in tumor cells and suppression of Treg cell proliferation, combined CDK4/6 degradation therapy with immunotherapy. The predictive role of PD-L1 in immunotherapy for solid tumors has been affirmed by several studies. It is generally accepted that high expression of PD-L1 is associated with satisfactory immune response and clinical benefits from ICBs treatment.^50,51^ In addition, an increased recruitment of immunosuppressive Treg cells lead to a reduced immune response against tumor cells. Several studies have shown that depletion of Treg cells in the TME could reverse ICBs therapy resistance.^52^ It’s noteworthy that Treg cells have a 1.6-fold higher uptake of PSRNs compared to CD8^+^ T cells (Fig. 5h). This observation suggested a potential exploration of the impact of nanoparticles’ size on the uptake efficiency by immune cells. Moreover, Rb1 expression (a key molecular in downstream of CDK4/6 pathway) in Treg cells was 3.1-fold than that in CD8^+^ T cells.^53^ These all provided a molecular rationale for selective suppression of Treg proliferation (not that of CD8^+^ T cells).

Development of specialized delivery systems, such as the PSRNs in our study have the potential for addressing limited cellular internalization and undesirable tissue specificity of PROTACs via enhanced cellular uptake and degradation efficacy. Additionally, the potential for off-target effects remains a concern, as PROTACs can sometimes degrade proteins that are not intended targets.^54^ PSRNs with specific tumor distribution may have the potential to address the limitation. While our current research did not directly support this claim, it is promising to study the potential of PSRNs in addressing off-target effects of PROTACs. Furthermore, while PROTACs have shown efficacy in preclinical models, their human clinical trials are still under investigation, and there is a need for further research to fully understand their pharmacokinetics, toxicity, and overall therapeutic potential.^2^ Addressing these limitations through continued innovation in delivery strategies and combination therapies will be critical for realizing the full potential of PROTACs in cancer therapy.

In addition to CDK4/6 targeted PROTAC, PSRNs offer the flexibly to conjugate diverse PROTACs to meet diverse requirements and achieve efficacy against many tumors. Our strategy provides a precise delivery system for PROTACs, potentially addressing challenges related to difficult of delivery and absorption. Moreover, it offers a potential clinical strategy for the combination of PROTACs and immunotherapy and may pave the way for programmed PROTACs design.

## Methods

### Animals

BALB/c mice (male, 18–20 g) were purchased from Peking University Health Science Center (Beijing, China). CT26-bearing mice model was established by injecting 1 ×10^5^ CT26 cells in the right underarm of BALB/c mice subcutaneously. All animal experiments were performed under an Animal Ethics Committee of Peking University approved protocol (LA2020345).

### Preparation and characterizations of PSRNs and PNRNs

PEG-*b*-P(EPA-*r*-PROTAC) was dissolved in dimethyl sulfoxide (DMSO) and added into ddH_2_O under sonication. After removing DMSO by ultrafiltration, the PSRNs were finished as a yellow suspension. PNRNs was prepared under the same method except that PEG-*b*-P(EPA-*r*-PROTAC) was replaced with PEG-*b*-P(EH-*r*-PROTAC). For Cy5-labeled formulations, PEG-*b*-P(EPA)-Cy5 or PEG-*b*-P(EH)-Cy5 was added additionally during preparation.

The concentration of the preparation was adjusted to 1 mg/mL (calculated based on polymer concentration) for characterization. The particle size and zeta-potential were measured by Zetasizer Nano ZS (Malvern). UV absorption spectrum and fluorescence spectrum were analyzed by UV-Vis (Hitachi) and spectro fluorophotometer (Shimadzu), respectively. For transition pH of PSRNs, count rates at different pH were analyzed using Zetasizer Nano ZS.

### TEM analysis

TEM images in different medium were captured by transmission electron microscope (JEM 1400PLUS). 10 µL of formulation at a concentration of 1.0 mg/mL (calculated based on polymer concentration) was dropped onto copper grids treated with de-electrostatic methods. After standing at room temperature for 2 min, surface moisture was removed by clean filter paper gently. Subsequently, 5 µL of deionized water was used for rinsing, followed by the addition of 5 µL of 2% phosphotungstic acid solution (pH 7.0) for negative staining for 3 min. Surface stain was removed by clean filter paper, and the residual stain was dried using a cold air blower for approximately 30 s.

### In vitro release of PROTAC

Release of PROTAC in vitro from polymer, PSRNs and PNRNs were evaluated in different medium. Briefly, the formulations were dispersed in the medium (0.5 mL) and incubated at 37 °C with a shaking rate of 100 rpm. At predetermined time points, a sample of the medium (20 µL) was collected, then pre-cooled sodium azide solution (10 µL) and acetonitrile (70 µL) were added immediately. The concentration of the released PROTAC was examined by HPLC (Shimadzu. Mobile phase: A: ACN, 70%, B: H_2_O with 0.1% (*v/v*) ammonium hydroxide, 30%; flow rate: 1 mL/min; column temperature: 40 °C; measure wavelength: 359 nm).

### Western blot assay

PROTAC-induced protein CDK4/6 degradation in vitro was analyzed by western blot assay. Briefly, CT26, MDA-MB-231, MCF-7 and PANC-1 cells were seeded into 12-well plates at a density of 20 ×10^4^ cells per well. Following an overnight incubation at 37 °C, the culture medium was replaced with fresh medium containing either free PROTAC, PSRNs or PNRNs under the predetermined conditions. The concentration of PROTAC in PSRNs and PNRNs was quantified using UV spectrophotometry at 359 nm. For studying the PROTAC-mediated protein degradation, different concentrations of MG132 were used as the proteasome inhibitors co-incubated with the preparations for 16 h. CA-074Me was used as a cathepsin B inhibitor (20 μM) incubated for 4 h in advance. At the end of experiments, cell lysates were collected. The protein samples were separated by SDS-PAGE and transferred onto a PVDF membrane (Millipore). Then, the membranes were blocked with blocking buffer for 30 min at room temperature, and incubated with primary antibodies (anti-CDK4, 1:2000; anti-CDK6, 1:1000; anti-β-tubulin, 1:10000; anti-Rb phospho S807, 1:1000) at 4 °C overnight. After washing 3 times using tris buffered saline with 1% Tween-20, HRP-conjugated secondary antibodies (HRP-conjugated Goat Anti-Rabbit IgG, 1:10000; HRP-conjugated Goat Anti-Mouse IgG, 1:10000) were added and incubated with the membrane at room temperature for 1 h. Finally, after washing 6 times using tris buffered saline with 1% Tween-20, the membranes were imaged with a gel imager (Tanon). ImageJ was used for quantitative analysis.

### Cytotoxicity and cell cycle analysis in vitro

The in vitro cytotoxicity was evaluated using 3-(4,5-dimethylthiazol-2-yl)-2,5-diphenyltetrazolium bromide (MTT) assay. CT26, MDA-MB-231, MCF-7, PANC-1, HUVEC and L-929 cells were seeded into 96-well plates at a density of 1500 cells per well. Following an overnight incubation at 37 °C, the culture medium was replaced by fresh medium containing different PROTAC preparations at predetermined concentrations. After 72 h of incubation, MTT was added to each well for a subsequent 4 h incubation. Finally, a microplate reader (Thermo scientific) was used to measure the relative cell viabilities at an absorbance of 490 nm. The IC_50_ values were calculated by GraphPad.

For cell cycle analysis, CT26 cells were treated with different PROTAC preparations (PROTAC concentration of 1 μM) for 48 h. At the end of experiment, cells were collected and fixed with pre-cold 70% ethanol at 4 °C overnight. After washing with PBS, the cells were stained with propidium iodide at 37 °C for 30 min. Then, cells were measured by flow cytometer (Calibur 2, BD). The cell cycle was analyzed with the FlowJo (v10.6.2).

### Cellular uptake and penetration in vitro

The uptake of PSRNs and PNRNs at different pH values were quantified by flow cytometry (Calibur 2, BD). Briefly, CT26 cells were seeded into 12-well plates and incubated with Cy5-labeled preparations for predetermined time points at pH 7.4/6.6, respectively. The intracellular fluorescence intensity was then examined by flow cytometry. PSRNs taken in cells and cleaved intracellular PROTAC were measured by Liquid Chromatography-Mass Spectrometry (LC/MS) (AB SCIEX. Mobile phase: a gradient elution was performed using solvent A (0.1% formic acid in water, *v/v*) and solvent B (acetonitrile) at a flow rate of 0.4 mL/min). Concentration of PROTAC in cell homogenate after incubating with papain solution for 4 h was regarded as PSRNs internalized.

We further explored the uptake mechanism of PSRNs. Briefly, CT26 cells were plated onto 12-well plates and incubated with different inhibitors (Chlorpromazine and Dynasore, clathrin inhibitor; M-β-CD and Nystatin, caveolin inhibitor; Amiloride, macropinocytosis inhibitor; Cytochalasin D, cytoskeleton inhibitor; Hypertonic sucrose, cell membrane fluidity inhibitor; 4 °C, energy inhibitor) for 1 h. After incubation, medium was replaced with preparations included medium for another 2 h at pH 7.4/6.6, respectively. Pre-incubation with blank medium used as control. The intracellular fluorescence intensity was then examined by flow cytometry.

Tumor spheroids of MCF-7, CT26 and PANC-1 cells (used as high-, medium- and low-level permeability tumor model, respectively) were employed to evaluate penetration of different PROTAC preparations in vitro. Briefly, the 3D tumor spheroids were cultured in 48-well plate onto 2% agarose gel. Then, spheroids were incubated with Cy5-labeled preparations at pH 7.4 and 6.6 for 8 h. Z-stack images of spheroids were captured by confocal laser scanning microscopy (CLSM, Nikon). Fluorescence intensity profiles were analyzed using ImageJ.

### Pharmacokinetics and biodistribution in vivo

Health male BALB/c mice were employed to study the pharmacokinetics profiles of PSRNs and PNRNs. The mice were randomly divided into 2 groups (*n* = 5 per group), and administrated *i.v*. with Cy5-labeled preparations at a dose of 100 mg/kg, based on polymer concentration. At predetermined time intervals post-administration (0.033, 0.25, 0.5, 1, 3, 6, 12, 24, 48 and 72 h), blood samples were collected and centrifuged at 2000 rpm for plasma. Then an aliquot of plasma (20 µL) was mixed with acidified methanol (200 µL), vortexed for 10 min, then centrifuged again at 12000 rpm for 10 min to precipitate proteins. The supernatant was collected and analyzed using spectro fluorophotometer (λ_ex/em_ = 640/665 nm). Inject Dose (%) = (FI_Test_/FI_100%_) × 100%. Fluorescence intensity of indicated time points was recorded as FI_Test_. Fluorescence intensity of 0.033 min was regarded as FI_100%_. Pharmacokinetic parameters were analyzed by DAS 2.0.

The biodistribution of preparations in vivo was examined using CT26-bearing mice with tumor volumes of 150–200 mm^3^. Briefly, real-time fluorescence images of mice were collected at predetermined time points (1, 2, 4, 8, 12, 24 and 48 h) after *i.v*. administration using IVIS imaging system (IVIS Lumina Series III). At 24 and 48 h post-injection, mice were sacrificed. The major organs and tumor tissues were harvested for ex vivo fluorescence imaging. The relative fluorescence intensity derived from the images was used to semi-quantitative analysis of accumulation of preparations in tumors and biodistribution in major organs. To visualize penetration of preparations in tumor, the tumor tissues (24 h) were freezing-sectioned into slices of 10 µm. Followed by fixing with 4% paraformaldehyde and blocking with 5% FBS, slices were cultured with CD31 antibodies (1:600), Alexa Fluor-488 labeled secondary antibody (1:800) and anti-fade mounting medium (Hochest 33342 included). Then captured by automated pathology imaging system (Vectra Polaris, Akoya).

### Anti-tumor study in vivo

#### Therapeutic efficacy of PSNRs

CT26 colorectal cancer bearing mice were employed to assess anti-tumor efficacy of PSRNs in vivo. The mice with tumors in the range of 50–100 mm^3^ were randomly divided into six groups (*n* = 5 per group): Saline, PEG-*b*-P(EPA), free PROTAC, PSRNs, PSRNs + α-PD-1 and α-PD-1 alone. Dose: PROTAC of 5 mg/kg, α-PD-1 of 50 μg each. The treatment groups received intravenous injections of either PEG-*b*-P(EPA), free PROTAC, or PSRNs at a PROTAC dose of 5 mg/kg on days 0, 3, 6, 9 and 12. Additionally, the PSRNs + α-PD-1 and the α-PD-1 alone group received intraperitoneal injections of α-PD-1 on days 1, 4, 7, 10 and 13. Tumor growth and body weight of mice were monitored every 2 days. The tumors, major organs and serums of mice were harvested at the end of the studies. The tumor burden should not exceed 10% of the animal’s body weight. Herein, CDK4/6 protein levels were evaluated by western blot assay with tumor lysate. In addition, tumors and organs were fixed and stained with H&E for investigating anti-tumor efficacy and primary safety evaluation.

The tumor volume was calculated with formula: V = Length × Width × Width/2.

#### Survival experiment of PSNRs

We employed CT26-bearing mice for survival experiment. Mice with tumor volumes of 50–100 mm^3^ were randomly divided into six groups (*n* = 5 per group): Saline, PEG-*b*-P(EPA), free PROTAC, PSRNs, PSRNs + α-PD-1 and α-PD-1. Dose: PROTAC of 5 mg/kg, α-PD-1 of 50 μg each. Experimental schedule was described above. Tumor growth and body weight of mice were monitored every 2 days.

After treatment, the condition of the mice was monitored continuously. The mice were euthanized when the tumor volume reached 2000 mm^3^.^32,55^ The survival time of mice in each group was recorded for survival curve.

### Protein degradation efficacy in vitro and in vivo

#### Wash-out assay in vitro

To evaluate protein degradation efficiency of PSRNs in different medium, wash-out assay was used. Briefly, CT26 cells were plated onto 12-well plates and then incubated with different PROTAC preparations at pH 7.4 and 6.6 (PROTAC of 1 μM). After incubation for 16 h, PROTAC-containing medium was replaced with regular medium. Cells were cultured continuously. Protein expression level in cell lysate was then analyzed by western blot assay at predetermined time points. ImageJ was used for quantitative analysis.

#### Protein level in vivo post-single administration

CT26-bearing mice were employed to investigate changes of protein expression at tumor site post-single administration. Briefly, CT26 bearing mice with tumor volume of about 200 mm^3^ were randomly divided into 2 groups (free PROTAC, PSRNs, *n* = 12 per group). Mice were sacrificed after 12, 24, 48 and 72 h of injection of different preparations. Tumors were extracted for western blot assay to evaluate changes of CDK4/6 protein expression (tumors from untreated mice used as negative control). ImageJ was used for quantitative analysis.

CDK4/6 protein degradation percentages (%) were calculated as formula below:

$${\rm{Protein\; degradation}}\, \% =({{\rm{A}}}_{100 \% }-{\rm{AUC}})/{{\rm{A}}}_{100 \% }\times 100 \%$$

Based on data quantified by ImageJ, the relative protein expression level change curves vs. time were plotted. Relative protein expression level of 100% used as initial protein expression level, and plotted a straight line. A_100%_ represents area under the straight. AUC represents the area under the curve of protein expression changes.

Further, tumor tissues were freezing-sectioned into slices of 10 µm. Followed by fixing with 4% paraformaldehyde and blocking with 5% FBS, slices were cultured with CDK4/6 antibodies, fluorescein labeled secondary antibody and Hochest 33342, in sequence. Then captured by CLSM to visualize CDK4/6 expression level in tumor.

### Expression level of PD-L1 in vitro

Briefly, CT26 cells were incubated with different PROTAC preparations for 48 h. At the end of experiment, cells were collected to investigate PD-L1 protein levels by flow cytometer and western blot assay. IFN-γ and palbociclib treated group were used as positive control.

### In Vivo immune response analysis

#### Flow cytometry for immune cell in vivo

Spleen and lymph nodes: single cell suspensions were obtained by mechanical digestion.

Tumor tissues: single cell suspensions were obtained by enzyme digestion. Briefly, tumor tissues were firstly cut into pieces, then incubated with enzyme digestion solution (0.5 mg/mL collagenase IV, 0.2 mg/mL DNase and 0.2 mg/mL hyaluronidase in 1640 culture medium containing 5% FBS and 1% penicillin-streptomycin) at 37 °C for 30 min. Finally, single lymphocyte suspensions were separated by lymphocytes isolation kits.

One million cells per condition were stained with fluorochrome conjugated antibodies diluted in PBS for 30 min on ice. For intracellular stain, cell suspensions should be fixed and permeabilizated with 4% paraformaldehyde and permeabilization buffer. Cellometer (Nexcelom) was used to determine the number of cells. Flow cytometry was performed on LSRFortessa (BD Biosciences), and data were analyzed using FlowJo (10.6.2). Flow cytometry gating strategies were performed in Supplementary Fig. 24.

#### Uptake of PSRNs by immune cells post *i.v*. administration

To evaluate the uptake of PSRNs by immune cells in CT26 bearing mice. Briefly, CT26-bearing mice with tumor volume of about 150 mm^3^ were randomly divided into 2 groups (PSRNs and PNRNs, *n* = 3 per group). After intravenous administration of Cy5-labeled nanoparticles, mice were sacrificed. Tumors were collected and prepared into single lymphocyte suspension. The single-cell suspensions were incubated with appropriate-conjugated antibodies for 30 min on ice. After washing twice with cold PBS, suspensions were fixed. PBS washing was processed prior to flow cytometry analysis. Cy5 signals in Treg cells and CD8^+^T cells were collected.

#### Remodeling immunosuppression environment post in situ administration

Inhibitions of CDK4/6 suppress the proliferation of Treg cells. CT26 bearing mice with tumor volume around 150 mm^3^ were randomly divided into 4 groups (Saline, free PROTAC, PSRNs and palbociclib; Dose: PROTAC of 2 mg/kg; *n* = 5 per group). Mice were injected intratumorally with saline, free PROTAC, PSRNs and palbociclib (as positive control), respectively, every 2 days. Tumor growth and body weight of mice were monitored every 2 days. After 5 cycles, mice were sacrificed and tumor tissues were collected for immune-environment analysis. Tumor infiltrating lymphocytes (TILs) were prepared into single-cell suspension using mice lymphocytes isolation kit. The single-cell suspensions were incubated with fluorophore-conjugated antibodies: CD45-Pacific blue, CD3-FITC, CD8a-APC, CD4-PE/Cy7, and CD25-PerCP/Cy5.5 for 30 min on ice. After washing with cold PBS 2 times, suspensions were fixed and permeabilizated, and then incubated with Foxp3-PE antibody at 4 °C overnight. PBS washing was processed prior to flow cytometry analysis. Lymph nodes and spleens were also collected, stained and analyzed mentioned above. TGF-β and IFN-γ levels in serum were evaluated by ELISA kit.

#### In vivo immune response analysis post chem-immunotherapy

Tumors were harvested after chem-immunotherapy. TILs were prepared into single-cell suspension using mice lymphocytes isolation kit. Suspensions were stained by the same method above expect that adding one more fluorophore-conjugated intracellular antibodies (Granzyme B-BV421). PBS washing was processed prior to flow cytometry analysis. Lymph nodes and spleens were also collected, stained and analyzed by method mentioned above. TGF-β and IFN-γ levels in serum and tumor homogenate were evaluated by ELISA kit.

### Statistical analysis

The GraphPad Prism 8.0 was used for the statistical analyses. Unpaired t-test and One-way analysis of variance (ANOVA) were used for statistical comparison. Log-rank (Mantel–Cox) test was used for the statistical comparison of the survival study.

## Supplementary information

Supplementary information

Chemical Structures

Raw data of western blot assays

## Data Availability

All data used in this paper are available from the corresponding author by reasonable requirements.
