# Supplementary material for: Sequential responsive nano-PROTACs for precise intracellular delivery and enhanced degradation efficacy in colorectal cancer therapy
Source: Signal Transduct Target Ther. 2024 Oct 18;9:275. doi: 10.1038/s41392-024-01983-1 (PMC11486899; doi:10.1038/s41392-024-01983-1)
Supplement: Supplementary file 1 — Supplementary information [file 41392_2024_1983_MOESM1_ESM.docx]

Supplementary Materials for

Sequential responsive nano-PROTACs for precise intracellular delivery and enhanced degradation efficacy in colorectal cancer therapy

Liuqing Yang^1^, Ye Yang^1^, Jing Zhang, Minghui Li, Long Yang, Xing Wang, Meifang Chen, Hua Zhang, Bing He, Xueqing Wang, Wenbing Dai*, Yiguang Wang*, Qiang Zhang*

Correspondence to: [daiwb@bjmu.edu.cn](mailto:daiwb@bjmu.edu.cn); [yiguang.wang@pku.edu.cn](mailto:yiguang.wang@pku.edu.cn); [zqdodo@bjmu.edu.cn](mailto:zqdodo@bjmu.edu.cn)

**This PDF file includes:**

Materials and Methods

Figures. S1 to S24

Tables S1 to S6

Materials and Methods

Materials

Palbociclib were purchased from Energy-Chemical (China). (2*S*,4*R*)-1-((*S*)-2-amino-3,3-dimethylbutanoyl)-4-hydroxy-N-((*S*)-1-(4-(4-methylthiazol-5-yl)phenyl) ethyl)pyrrolidine-2-carboxamide (VHL) was purchased from Bide Pharmatech Co., Ltd.. Synthesis materials and resins for tetrapeptide were purchased from GL Biochem (Shanghai) Ltd.. All other synthesis materials were purchased from Aladdin (China). Acetonitrile (ACN, HPLC grade) and methyl alcohol (MeOH, HPLC grade) were purchased from Thermo Scientific. Petroleum ether, absolute ethyl alcohol (EtOH), tetrahydrofuran (THF) and ethyl acetate (EtOAc) were purchased from TGREAG. Dimethylformamide (DMF), dimethylsulfoxide (DMSO), dichloromethane (DCM), trifluoroacetic acid (TFA) and tributylphosphine were purchased from J&K Scientific Ltd.. MG132 (proteasome inhibitor), CA-074Me (cathepsin B inhibitor) and other inhibitors were obtained from MedChemExpress. Papain and lymphocytes isolation kits were purchased from Beijing Solarbio Science & Technology Co,. Ltd.. IFN-γ was purchased from Yeasen Biotechnology (Shanghai) Co., Ltd.. Anti-CDK4 antibody (ab199728), anti-CDK6 antibody (ab241554), anti-Rb (phospho S807) antibody (ab184796), anti-PD-L1 antibody (ab213480), Alexa Fluor 488-conjugated goat anti-rabbit IgG (ab150077), Alexa Fluor 488-conjugated goat anti-mouse IgG (ab150113) were all purchased from Abcam. Anti-β-tubulin antibody (66240-1-Ig) and anti-CD31antibody (66065-2-Ig) were purchased from Proteintech. Anti-VHL antibody (AF6292) was purchased from Affinity Biosciences Ltd.. Anti-GAPDH antibody (AF1186), HRP-conjugated goat anti-rabbit IgG (A0208), HRP-conjugated goat anti-mouse IgG (A0216), anti-fade mounting medium (Hochest 33342 included) and all other reagents for western blot assay were purchased from Beyotime Biotechnology. Pacific Blue anti-mouse CD45 (103125), APC/Cy7 anti-mouse CD45 (103115), FITC anti-mouse CD3 (100203), PE/Cy7 anti-mouse CD4 (116015), APC anti-mouse CD8a (100712), PerCP-Cy5.5 anti-mouse CD25 (102029), PE anti-mouse CD25 (102007), BV405 anti-mouse Granzyme B (396413) were purchased from Biolegend. PE-Foxp3 antibody (12-5773-82), permeabilization buffer (00-8333-5), and ELISA kits for TGF-β and IFN-γ detection were purchased from Invitrogen. Roswell Park Memorial Institute (RPMI) 1640 medium, Dulbecco's Modified Eagle's Medium (DMEM) and penicillin-streptomycin were purchased from MACGENE (Beijing) Biotechnology Ltd.. Fetal bovine serum (FBS) was purchased from Gemibio.

Chemical characterization

Melting points were determined on a Buchi M-560 melting point apparatus. Electrospray ionization-mass spectrometry spectrum (ESI-MS) was conducted with mass spectrometer (API 4000, AB Sciex) equipped with a standard ESI source. High-resolution mass spectrometry (HRMS) was recorded on Thermo Q Exactive HF-X mass spectrometer (Thermo Scientific) equipped with an ESI-source. Proton nuclear magnetic resonance spectrum (^1^H-NMR) and carbon-13 nuclear magnetic resonance spectrum (^13^C-NMR) were conducted by 600 MHz NMR spectrometer (AVANCE NEO 600 MHz spectrometer, Bruker). Ultraviolet-visible (UV-Vis) spectrum and fluorescence spectra were analyzed on U2600 dual beam spectrophotometer (Shimadzu) and RF-6000 spectro fluorophotometer (Shimadzu), respectively. High performance liquid chromatography (HPLC) chromatogram was measured by HPLC using acetonitrile/H_2_O as the eluent (Shimadzu). Purification of polypeptide was performed on preparative liquid chromatography (1525 binary HPLC pump, Waters). Gel permeation chromatography (GPC) analysis was carried out on a E2695 Gel Penetrate Chromatogram (Waters) equipped with a PLgel MIXED-C column (Agilent). 10 mg/mL of samples were eluted by tetrahydrofuran at a flow rate of 1 mL per minute and detected by differential refraction detector.

Cell lines

Mouse colorectal cancer CT26 cell line, mouse fibroblast L-929 cell line, human breast cancer MCF-7 cell line, human breast cancer MDA-MB-231 cell line, human pancreatic cancer PANC-1 cell line and human umbilical vein endothelial (HUVEC) cell line were obtained from Cell Resource Center, Peking Union Medical College. Herein, CT26 and MCF-7 cells were maintained in RPMI 1640 medium with 10% fetal bovine serum (FBS) and 1 % penicillin-streptomycin. Other cells were cultured in Dulbecco’s modified Eagle media medium (DMEM) with 10% FBS and 1 % penicillin-streptomycin. All cells were cultured at 37 °C under 5% CO_2_ atmosphere.

Synthesis of CDK4/6 targeted PROTAC^1^

Synthesis of ethyl 8-(4-(6-((6-acetyl-8-cyclopentyl-5-methyl-7-oxo-7,8-dihydropyrido [2,3-d] pyrimidin-2-yl)amino)pyridin-3-yl)piperazin-1-yl)octanoate (**Compound 1**)

Ethyl 8-bromooctanoate (336 mg, 1.34 mmol), potassium carbonate (463 mg, 3.35 mmol) and potassium iodide (185 mg, 1.12 mmol) were added to stirred solution of palbociclib (500 mg, 1.20 mmol) in DMF (25 mL). The reaction mixture was stirred at 80 °C for 16 hours under N_2_ atmosphere, cooled to 25 °C, then extracted with EtOAc (3 × 25 mL). The combined organic phases were washed with brine (50 mL), dried over Na_2_SO_4_ and concentrated *in vacuo*. The crude material was purified by Silica gel column chromatography (EtOAc / Petroleum ether 3:1) to yield the title compound as a faint yellow solid. Weight: 310 mg. Yield: 62%. MS (ESI): *m/z* [M+H]^+^ 618.6.

Synthesis of 8-(4-(6-((6-acetyl-8-cyclopentyl-5-methyl-7-oxo-7,8-dihydropyrido [2,3-d] pyrimidin-2-yl)amino)pyridin-3-yl)piperazin-1-yl)octanoic acid (**compound 2**)

Lithium hydroxide (120 mg, 5.0 mmol) was added to stirred suspension of compound 1 (310 mg, 0.50 mmol) in THF (5.0 mL), EtOH (2.5 mL) and H_2_O (2.5 mL) at 0 °C. The reaction mixture was stirred at 25 °C overnight, concentrated in vacuo, then ice water (15 mL) was added. The mixture was acidified to pH 5-6 using 1 M HCl solution. The resultant precipitate was collected by centrifugation and washed with ice water (2 × 10 mL). Further yield compound 2 by freeze-drying a yellow solid used for further reaction without purification. Weight: 280 mg. Yield: 95 %. MS (ESI): *m/z* [M+H]^+^ 589.3.

Synthesis of (2*S*,4*R*)-1-((*S*)-2-(8-(4-(6-((6-acetyl-8-cyclopentyl-5-methyl-7-oxo-7,8-dihydropyrido[2,3-*d*]pyrimidin-2-yl)amino)pyridin-3-yl)piperazin-1-yl)octanamido)-3,3-dimethylbutanoyl)-4-hydroxy-N-((*S*)-1-(4-(4-methylthiazol-5-yl)phenyl)ethyl)pyrrolidine -2-carboxamide (**PROTAC**)

2-(7-Azabenzotriazol-1-yl)-N, N, N', N'-tetramethyluronium hexafluorophosphate (HATU, 216 mg, 0.570 mmol) was added to a stirred solution of crude compound 2 (280 mg, 0.475 mmol) in DMF (10 mL) at 0 °C. The mixture was stirred at 25 °C for 15 min, followed by addition of N, N-Diisopropylethylamine (DIPEA, 330 μL, 1.9 mmol) and VHL (257 mg, 0.570 mmol). The reaction solution was stirred at 25 °C overnight. Then extracted with DCM (3 × 25 mL). The combined organic phases were washed with brine (50 mL), dried over Na_2_SO_4_ and concentrated *in vacuo*. The crude product was purified by column chromatography (DCM / MeOH 50:1) to yield the PROTAC as a yellow solid. Weight: 314 mg. Yield: 65%. Melting point: 206.5 ℃. MS (ESI): *m/z* [M+H]^+^ 1016.8. ^1^H-NMR (600 MHz, DMSO-*d_6_*) δ 10.09 (s, 1H), 8.98 (s, 1H), 8.95 (s, 1H), 8.37 (d, 1H), 8.05 (d, 1H), 7.84 (d, 1H), 7.79 (d, 1H), 7.47-7.42 (m, 3H), 7.38-7.37 (m, 2H), 5.82 (quin, 1H), 5.10 (d, 1H), 4.92 (quin, 1H), 4.52 (d, 1H), 4.42 (t, 1H), 4.30-4.26 (m, 1H), 3.63-3.57 (m, 2H), 3.17-3.12 (m, 4H), 2.45 (s, 3H), 2.42 (s, 3H), 2.32-2.29 (m, 5H), 2.27-2.22 (m, 3H), 2.13-2.08 (m, 1H), 2.03-1.98 (m, 1H), 1.91-1.85 (m, 2H), 1.81-1.74 (m, 3H), 1.60-1.55 (m, 2H), 1.54-1.41 (m, 5H), 1.39-1.36 (m, 3H), 1.33-1.22 (m, 7H), 0.94 (s, 9H). ^13^C-NMR (151 MHz, DMSO-*d_6_*) δ: 202.46, 172.06, 170.62, 169.62, 160.76, 158.57, 158.26, 154.76, 151.47, 147.75, 144.65, 144.23, 143.49, 142.10, 135.28, 131.11, 129.69, 129.20, 128.82, 126.38, 124.62, 115.15, 106.54, 68.75, 58.54, 57.87, 56.33, 56.25, 52.89, 52.57, 48.34, 40.05, 37.73, 35.19, 34.88, 31.31, 28.69, 28.65, 27.55, 26.87, 26.45, 26.24, 25.39, 25.11, 22.43, 15.98, 13.62. HRMS (ESI): *m/z* [M + H]^+^ calcd for C_55_H_73_N_11_O_6_S, 1016.55388; found 1016.55300. HPLC purity: 97.2%.

Synthesis of synthesis of monomer MA-GFLG-PROTAC

Synthesis of N-methacryloylglycylphenylalanylleucylglycine (**MA-GFLG(OH)**)

Solid phase synthesis method was used for synthesis of monomer MA-Gly-Phe-Leu-Gly (OH), MA-GFLG (OH) for short. The tetrapeptide GFLG was recognition site for cathepsin B. The methacryloyl (MA) was used for reversible addition-fragmentation chain transfer (RAFT) polymerization method.^2^ Synthesis route was shown in supplementary Fig. 3. The crude product was purified by prep-HPLC with 10-50 % ACN aqueous gradient elution for 15 min. The target fractions were combined and concentrated using Freeze-drying to afford MA-GFLG (OH) as a white flocculent solid. Weight: 381 mg. Yield: 83 %. MS (ESI): *m/z* [M+H]^+^ 461.9. ^1^H-NMR (600 MHz, DMSO-*d_6_*) δ 12.51 (s, 1H), 8.13 (t, 1H), 8.09 (d, 1H), 8.05 (t, 1H), 7.99 (d, 1H), 7.35-6.96 (m, 5H), 5.69 (s, 1H), 5.40-5.32 (m, 1H), 4.56-4.49 (m, 1H), 4.33 (q, 1H), 3.79-3.67 (m, 3H), 3.63-3.57 (m, 1H), 3.06-2.98 (m, 1H), 2.83-2.75 (m, 1H), 1.84 (s, 3H), 1.64-1.56 (m, 1H), 1.55-1.40 (m, 2H), 0.89 (dd, 3H), 0.84 (d, 3H).

Synthesis of monomer **MA-GFLG-PROTAC**

PROTAC (314 mg, 0.309 mmol), MA-GFLG (OH) (213 mg, 0.463 mmol), 4-Dimethylaminopyridine (DMAP, 15 mg, 0.123 mmol) and DIPEA (213 μL, 1.9 mmol) were dissolved in DCM. 1-(3-Dimethylaminopropyl)-3-ethylcarbodiimide hydrochloride (EDCI, 118 mg, 0.618mmol) dissolved in DCM was added dropwise to reaction mixture at 0 °C. Then, the reaction system was stirred at 25 °C overnight. Then, DCM was removed by rotary evaporator and crude product was dissolved in EtOAc followed by washing with NH_4_Cl, NaHCO_3_ and NaCl saturated solutions, respectively. Then, the organic phase was dried over MgSO_4_ and evaporated to remove EtOAc. The crude product was purified by prep-HPLC with 50-80% ACN aqueous gradient elution for 25 min. The target fractions were combined and concentrated using freeze-drying to afford MA-GFLG-PROTAC as a yellow solid. Weight: 252 mg. Yield: 56 %. Melting point: 147.6-152.3 ℃. MS (ESI): *m/z* [M+H]^+^ 1459.0. ^1^H-NMR (600 MHz, DMSO-*d_6_*) δ 10.08 (s, 1H), 8.98 (s, 1H), 8.94 (s, 1H), 8.41 (d, 1H), 8.20 (t, 1H), 8.12 (t, 1H), 8.08 (d, 1H), 8.04 (d, 1H), 7.98 (d, 1H), 7.86-7.82 (m, 2H), 7.47-7.44 (m, 1H), 7.43-7.41 (m, 2H), 7.39-7.36 (m, 2H), 7.22-7.20 (m, 4H), 7.18-7.14 (m, 1H), 5.81 (quin, 1H), 5.69 (s, 1H), 5.35 (t, 1H), 5.24-5.27 (m, 1H), 4.91 (quin, 1H), 4.56-4.51 (m, 1H), 4.47 (t, 1H), 4.37-4.32 (m, 2H), 3.94 (d, 1H), 3.82-3.72 (m, 4H), 3.62-3.58 (m, 1H), 3.16-3.12 (m, 4H), 3.04-3.00 (m, 1H), 2.82-2.76 (m, 1H), 2.44 (s, 3H), 2.42 (s, 3H), 2.29-2.31 (m, 5H), 2.25-2.21 (m, 3H), 2.16-2.08 (m, 1H), 2.06-2.00 (m, 1H), 1.91-1.85 (m, 2H), 1.83 (s, 3H), 1.79-1.72 (m, 2H), 1.62-1.55 (m, 3H), 1.62-1.55 (m, 7H), 1.36 (d, 3H), 1.29-1.21 (m, 7H), 0.95 (s, 9H), 0.93 (s, 1H), 0.89 (d, 3H), 0.84 (d, 3H). ^13^C-NMR (151 MHz, DMSO-*d_6_*) δ: 202.46, 172.58, 172.41, 170.65, 169.87, 169.78, 169.36, 168.92, 167.63, 160.75, 158.57, 158.27, 154.75, 151.48, 147.76, 144.50, 144.24, 143.48, 142.08, 139.33, 137.63, 135.29, 131.08, 129.74, 129.27, 129.20, 128.84, 127.99, 126.33, 126.20, 124.61, 119.74, 115.17, 106.54, 73.58, 58.12, 57.85, 57.02, 53.69, 53.17, 52.87, 52.56, 50.85, 48.32, 47.78, 42.34, 40.96, 40.68, 40.06, 37.40, 34.72, 34.53, 31.31, 28.63, 27.54, 26.88, 26.40, 26.24, 25.40, 25.10, 24.03, 22.94, 22.43, 21.62, 18.49, 15.96, 13.62. HRMS (ESI): *m/z* [M + H]^+^ calcd for C_78_H_103_N_15_O_11_S, 1458.77550; found 1458.77396. [M + Na]^+^ calcd for C_78_H_103_N_15_O_11_S, 1480.75744; found 1480.75627. HPLC purity: 98.8%.

Synthesis of PEG-*b*-P(EPA), PEG-*b*-P(EH), PEG-*b*-P(EPA-*r*-PROTAC) and PEG-*b*-P(EH-*r*-PROTAC)

PEG-*b*-P(EPA-*r*-PROTAC) was synthesized via a RAFT method.^3^ Briefly, macro-chain transfer agent PEG-CTA (250 mg, 0.05 mmol), monomer EPA-MA^4^ (597 mg, 3.0 mmol) and MA-GFLG-PROTAC (73 mg, 0.15 mmol), and initiator 2,2'-Azobis(2-methylpropionamide) dihydrochloride (V50, 2.7 mg, 0.01 mmol) were dissolved with 2 ml dioxane and the mixture was kept at 50 ℃ for 48 h after three times of freezing-thawing cycle. The polymer product was successively dialyzed against DMF and distilled water to remove small molecules. Finally, the yellow powder was obtained by lyophilization. Similarly, the pH-nonresponsive polymer-drug conjugate PEG-*b*-P(EH-*r*-PROTAC) was synthesized by replacing EPA-MA with EH-MA. And the drug-free polymers were synthesized according to the procedures mentioned above by removing the monomer MA-GFLG-PROTAC.

Synthesis of PEG-*b*-P(EPA)-Cy5 and PEG-*b*-P(EH)-Cy5

Firstly, PEG-*b*-P(EPA) (1.0 eqv) was dissolved in MeOH (5 mL). Then, n-butylamine (10.0 eqv) and tributylphosphine (1.0 eqv) were added to reaction mixture under N_2_ atmosphere. After stirring for 4 h, Cy5-maleimide (0.5 eqv) was added at 25 °C under N_2_ atmosphere. After stirring for 48 h, the mixture was purified using ultrafiltration. The PEG-*b*-P(EH)-Cy5 was synthesized using the same method except that PEG-*b*-P(EPA) was replaced with PEG-*b*-P(EH). The Cy5 conjugation efficiency was determined by UV-Vis, according to standard curve of Cy5.

References

1 Steinebach, C. *et al.* Systematic exploration of different E3 ubiquitin ligases: an approach towards potent and selective CDK6 degraders. *Chem Sci*. **11**, 3474-3486, (2020).

2 Liu, J. H. *et al.* Prostate-Cancer-Targeted N-(2-Hydroxypropyl)methacrylamide Copolymer/Docetaxel Conjugates. *Macromol Biosci*. **12**, 412-422, (2012).

3 Du, H. L. *et al.* pH/Cathepsin B Hierarchical-Responsive Nanoconjugates for Enhanced Tumor Penetration and Chemo-Immunotherapy. *Adv Funct Mater*. **30**, 202003757, (2020).

4 Ma, X. P. *et al.* Ultra-pH-Sensitive Nanoprobe Library with Broad pH Tunability and Fluorescence Emissions. *J Am Chem Soc*. **136**, 11085-11092, (2014).

Figure. S1.


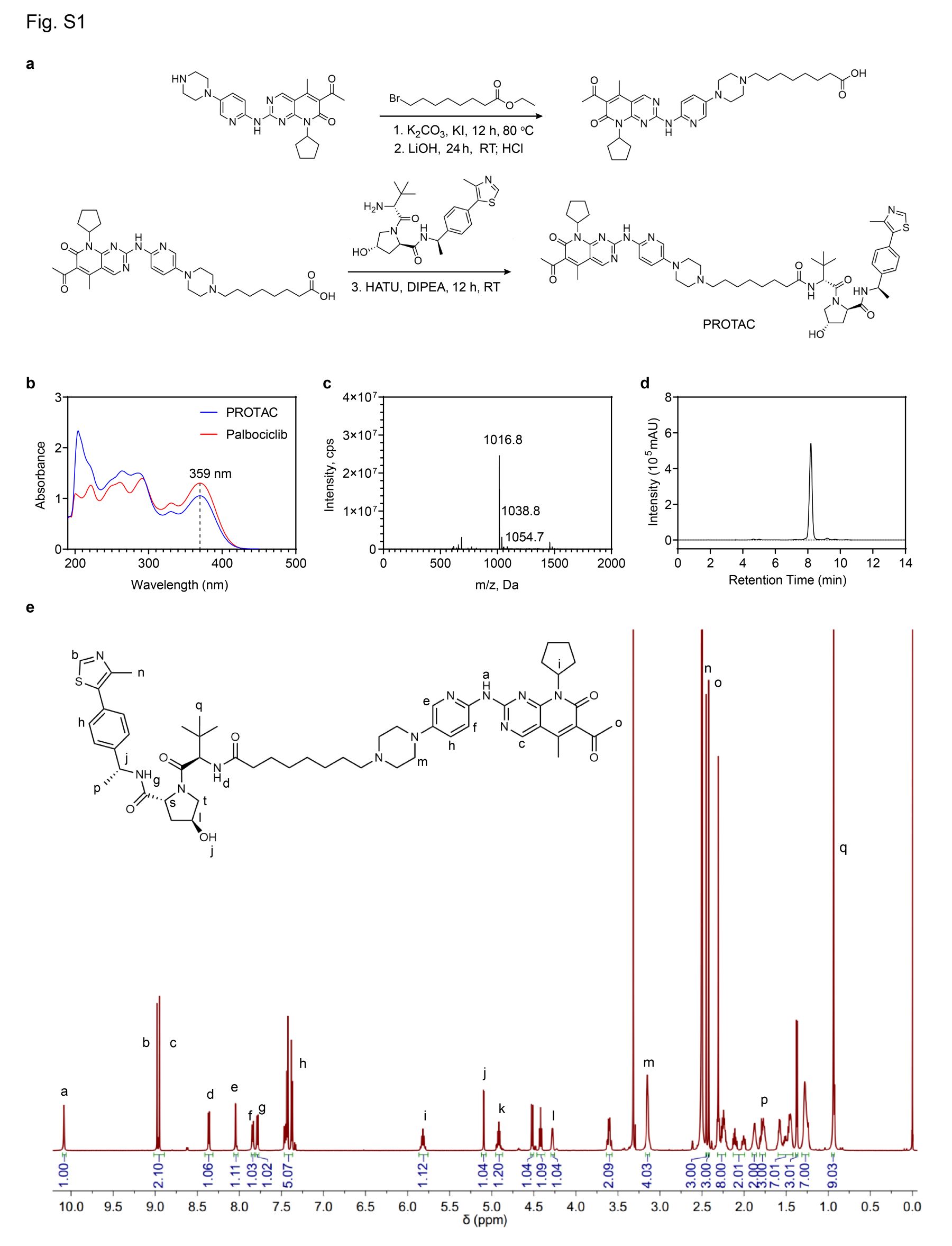


**Figure. S1.** The synthetic route (**a**), UV-Vis spectra (**b**), ESI-MS spectrum (**c**), found mass: [M+H]^+^=1016.8, [M+Na]^+^=1038.8, [M+K]^+^ =1054.7, HPLC chromatogram (**d**) and ^1^H-NMR spectrum (**e**) of PROTAC.

Figure. S2.


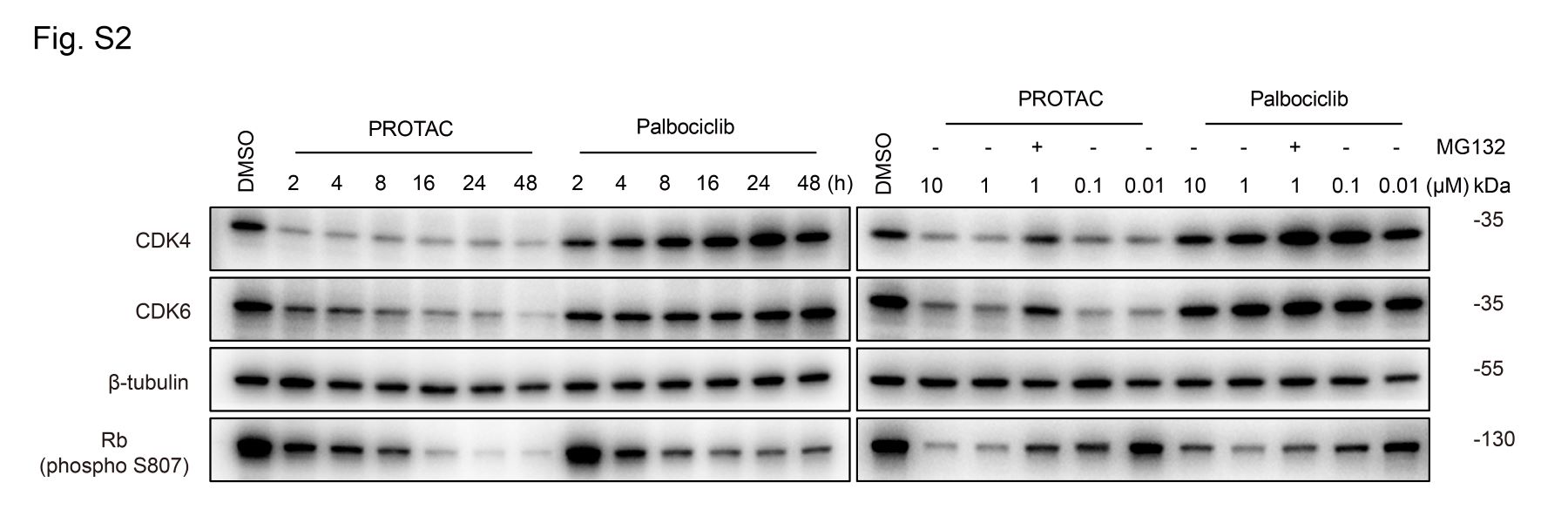


**Figure. S2**. Western blot assay of PROTAC-mediated CDK4/6 degradation and Rb (phospho S807) downregulation in MDA-MB-231 cells. Cells were treated with PROTAC and palbociclib (concentration of 1 μM) for indicated times (left). Cells were incubated with different concentration (μM) of PROTAC and palbociclib for 16 h (right, MG132 used as proteasome inhibitor at 0.5 µM). β-tubulin was used as loading control.

Figure. S3.


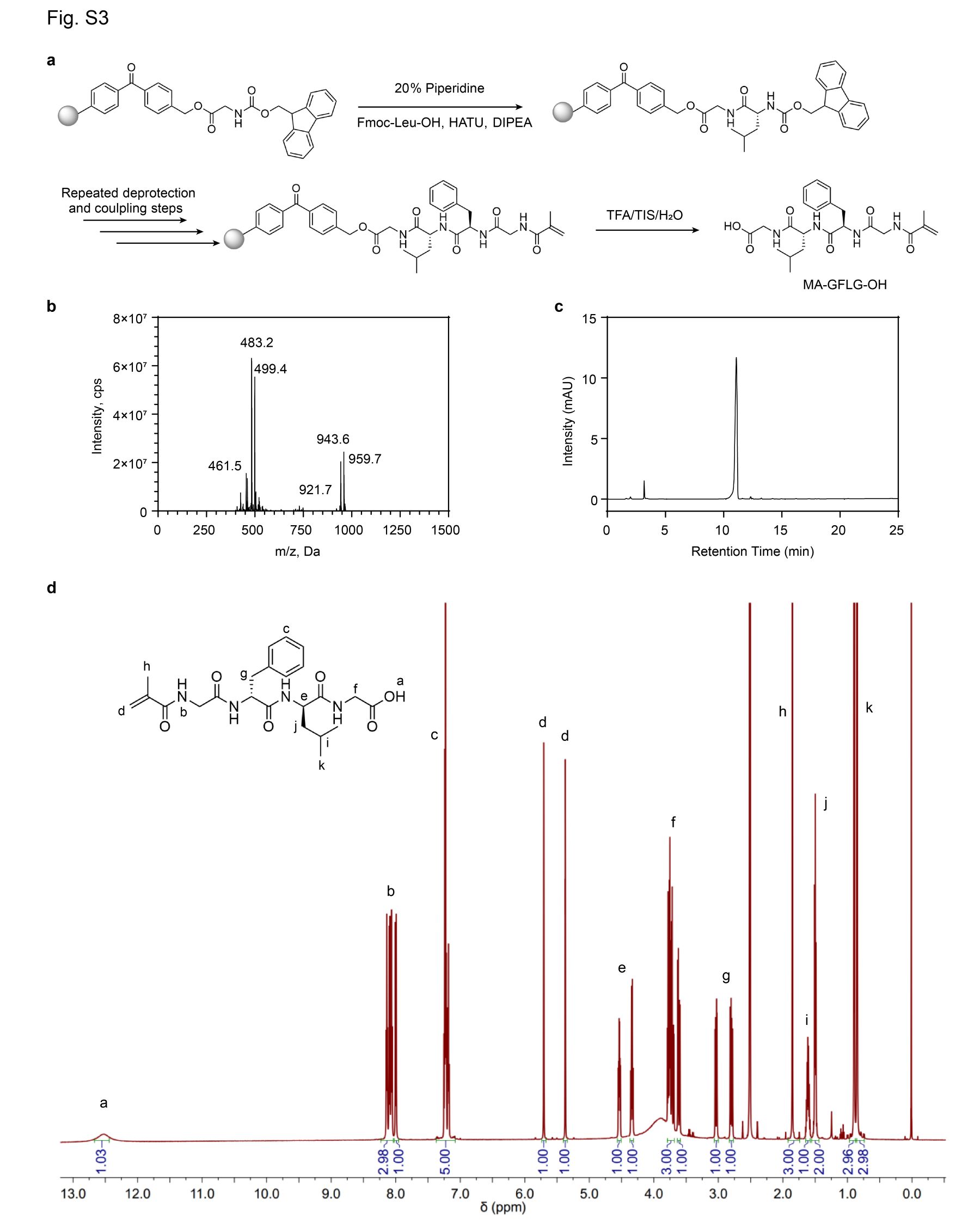


**Figure. S3**. The synthetic route (**a**), ESI-MS spectrum (**b**, found mass: [M+H]^+^=461.5, [M+Na]^+^=483.2, [M+K]^+^ =499.4, [2M+H]^+^=921.7, [2M+Na]^+^=943.6, [2M+K]^+^ =959.7), HPLC chromatogram (**c**) and ^1^H-NMR spectrum (**d**) of MA-GFLG-OH. The gray spherical shapes in (**a**) represent resins for solid phase synthesis. TIS, short for Triisopropylsilane.

Figure. S4.


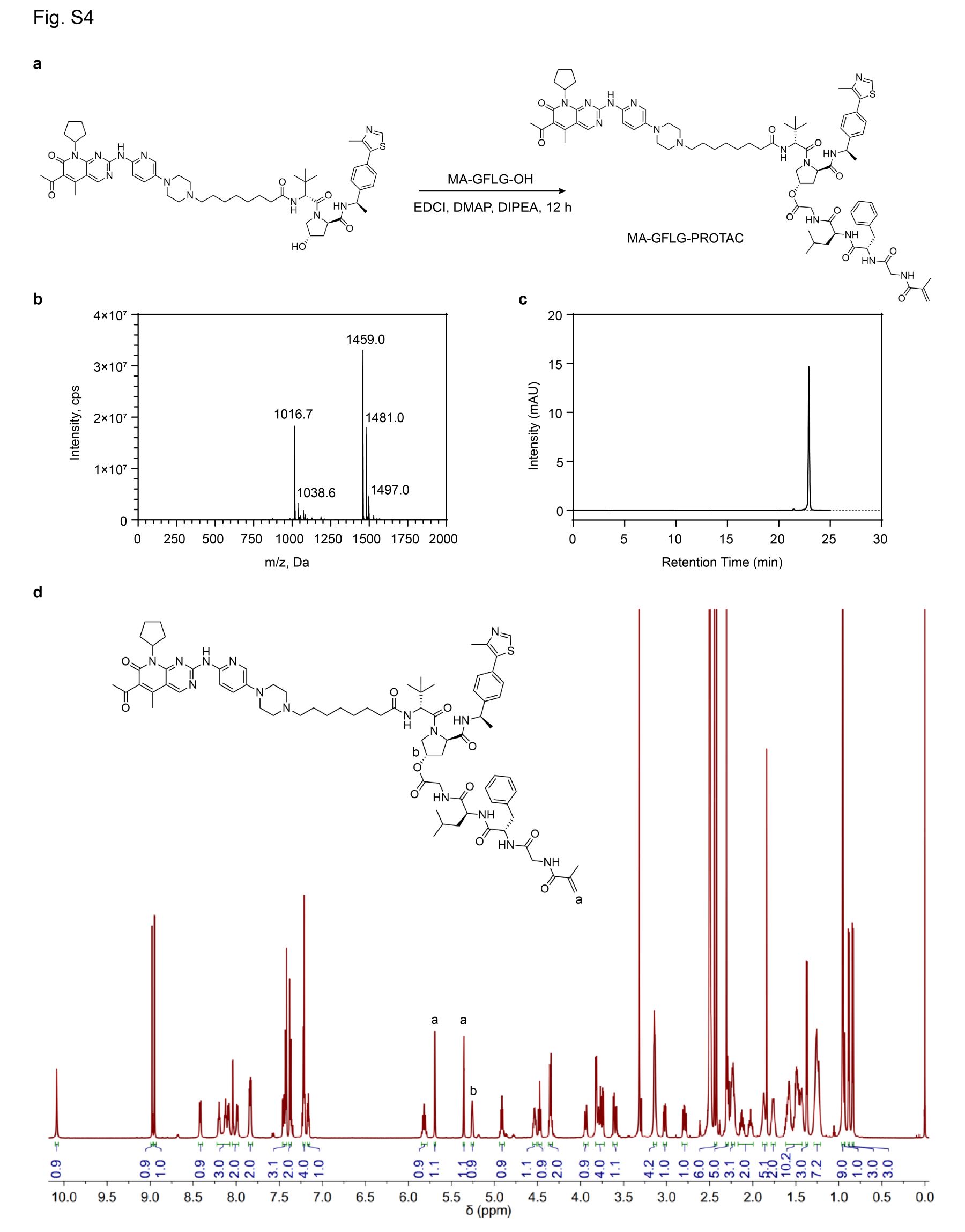


**Figure. S4.** The synthetic route (**a**), ESI-MS spectrum (**b**, found mass: [M+H]^+^=1459.0, [M+Na]^+^=1481.0, [M+K]^+^ =1497.0), HPLC chromatogram (**c**) and ^1^H-NMR spectrum (**d**) of MA-GFLG-PROTAC.

Figure. S5.


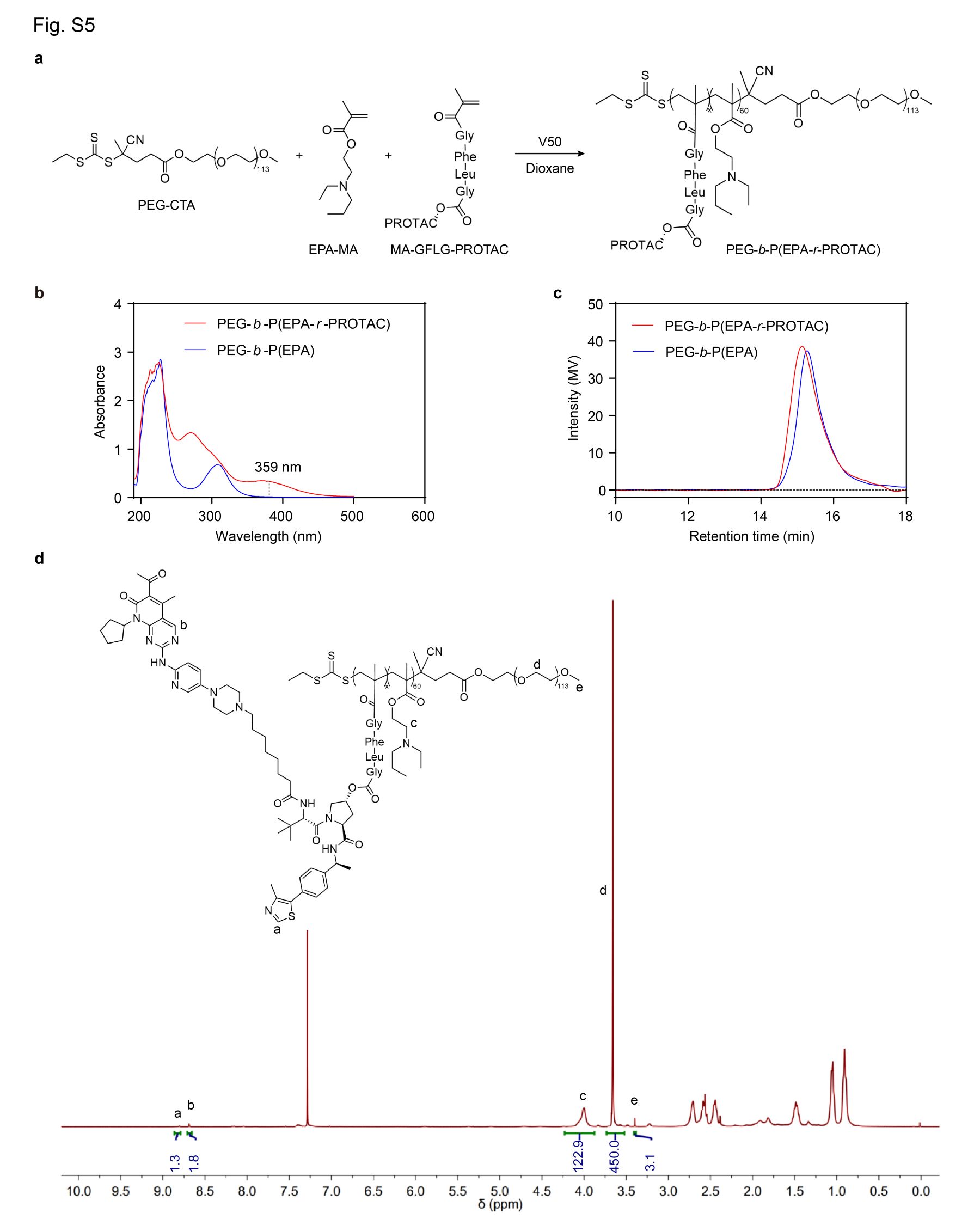


**Figure. S5**. The synthetic route (**a**) and ^1^H-NMR spectrum (**d**) of PEG-*b*-P(EPA-*r*-PROTAC) block copolymer. From the ^1^H-NMR spectrum, PROTAC content in polymer was calculated to be 7.2 %. The UV-Vis spectra (**b**) and GPC analysis (**c**) of PEG-*b*-P(EPA-*r*-PROTAC) and non-PROTAC conjugated PEG-*b*-P(EPA) polymer.

Figure. S6.


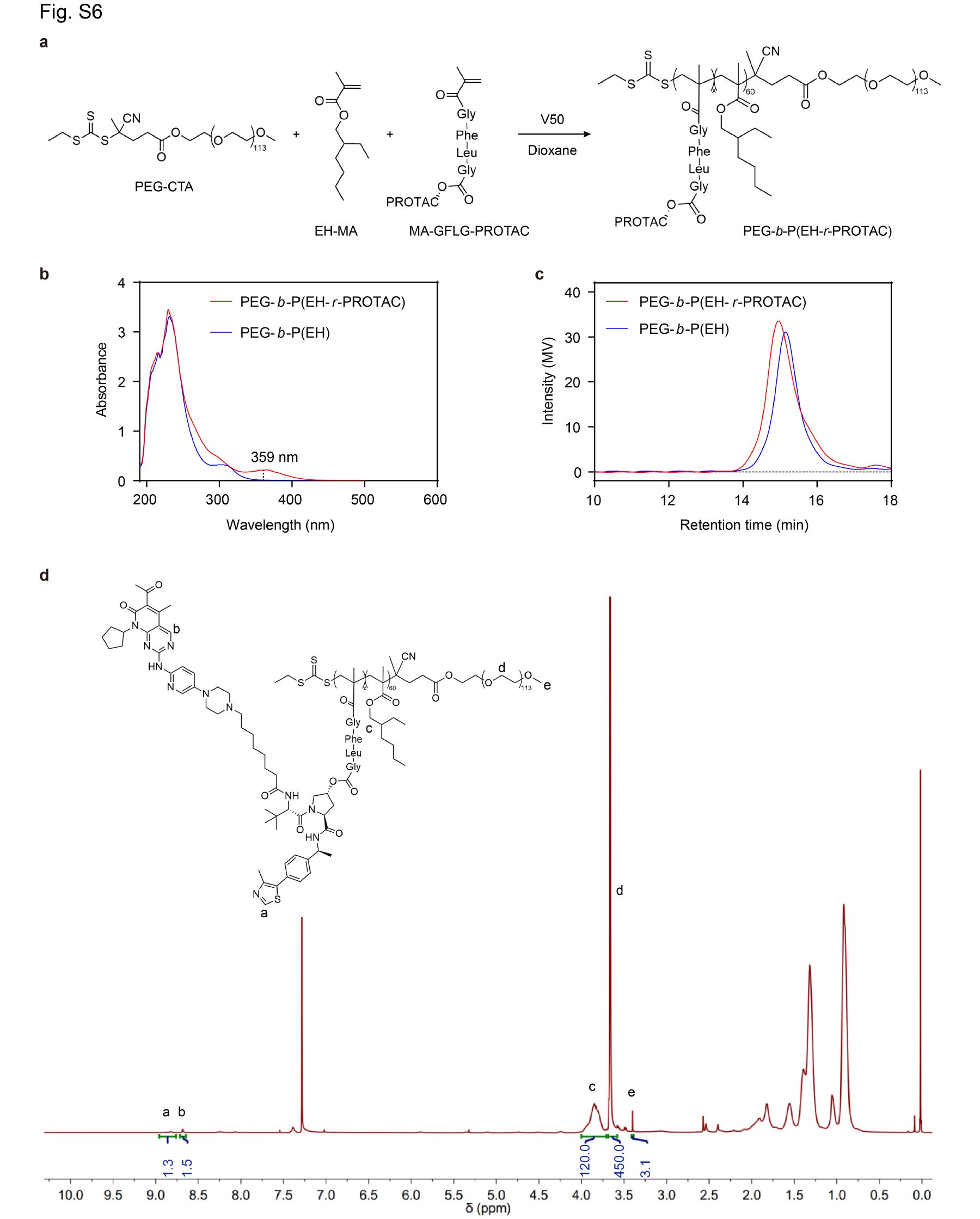


**Figure. S6**. The synthetic route (**a**) and ^1^H-NMR spectrum (**d**) of PEG-*b*-P(EH-*r*-PROTAC) block copolymer. From the ^1^H-NMR spectrum, PROTAC content in polymer was calculated to be 7.3 %. The UV-Vis spectra (**b**) and GPC analysis (**c**) of PEG-*b*-P(EH-*r*-PROTAC) and non-PROTAC conjugated PEG-*b*-P(EH) polymer.

Figure. S7.


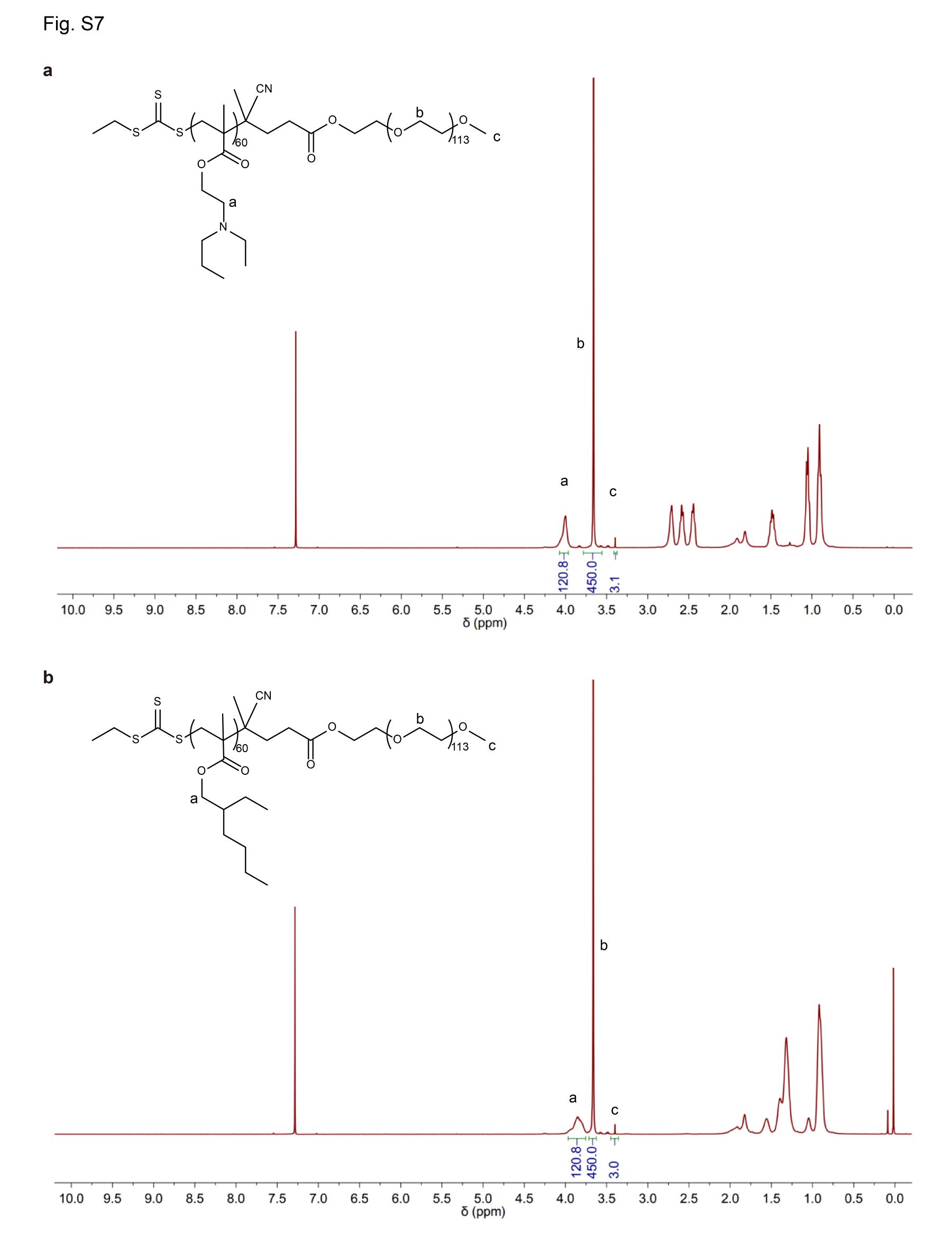


**Figure. S7.** The ^1^H-NMR spectrum of non-PROTAC conjugated block polymers, PEG-*b*-P(EPA) (**a**) and PEG-*b*-P(EH) (**b**).

Figure. S8.


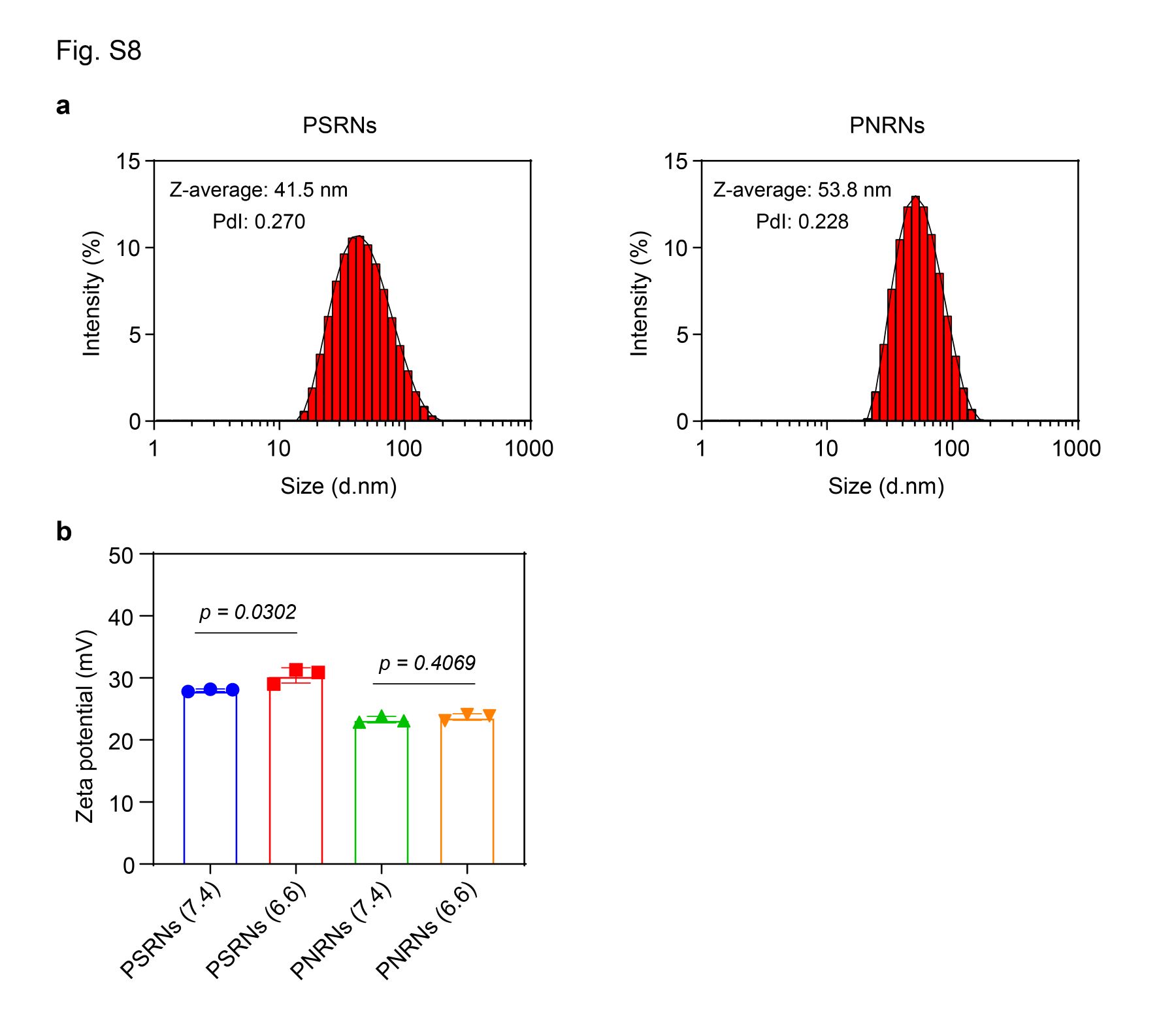


**Figure. S8.** **a**. Dynamic light scattering (DLS) profiles of PSRNs and PNRNs at pH 7.4. **b**. Zeta potentials of PSRNs and PNRNs at pH 7.4 and 6.6 (**b**). Mean ± SD, n = 3. All samples of 1 mg/mL, concentration based on polymer.

Figure. S9.


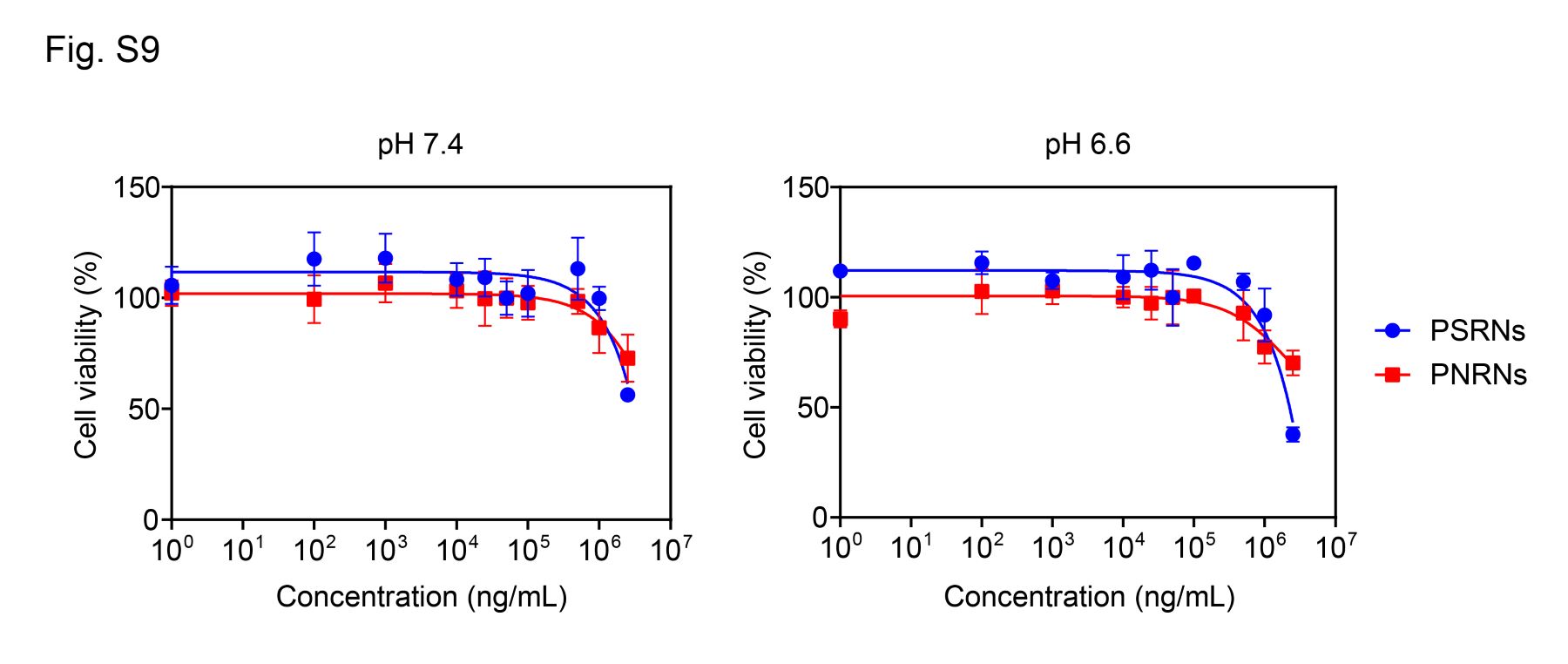


**Figure. S9.** Cell viabilities of CT26 cells after 72 h incubation with blank PSRNs and PNRNs (calculated based on concentration of polymer) at pH 7.4 and 6.6. Mean ± SD, n = 4.

Figure. S10.


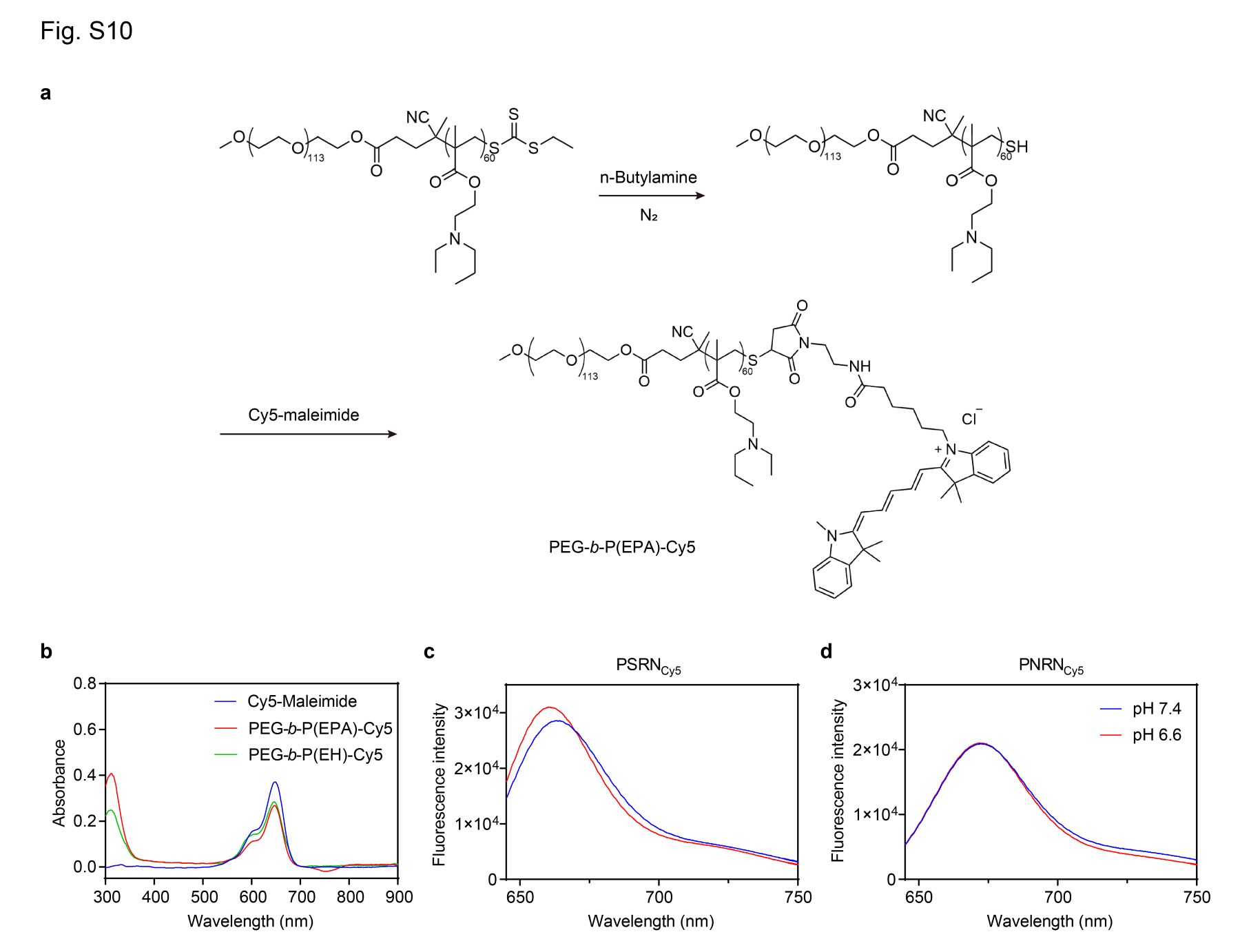


**Figure. S10.** The synthetic route (**a**) and UV-Vis spectra (**b**) of PEG-*b*-P(EPA)-Cy5 fluorescent polymer. Fluorescence emission spectra of PSRN_Cy5_ (**c**) and PNRN_Cy5_ (**d**) at pH 7.4 and pH 6.6. Each sample was excited at 640 nm for Cy5.

Figure. S11.


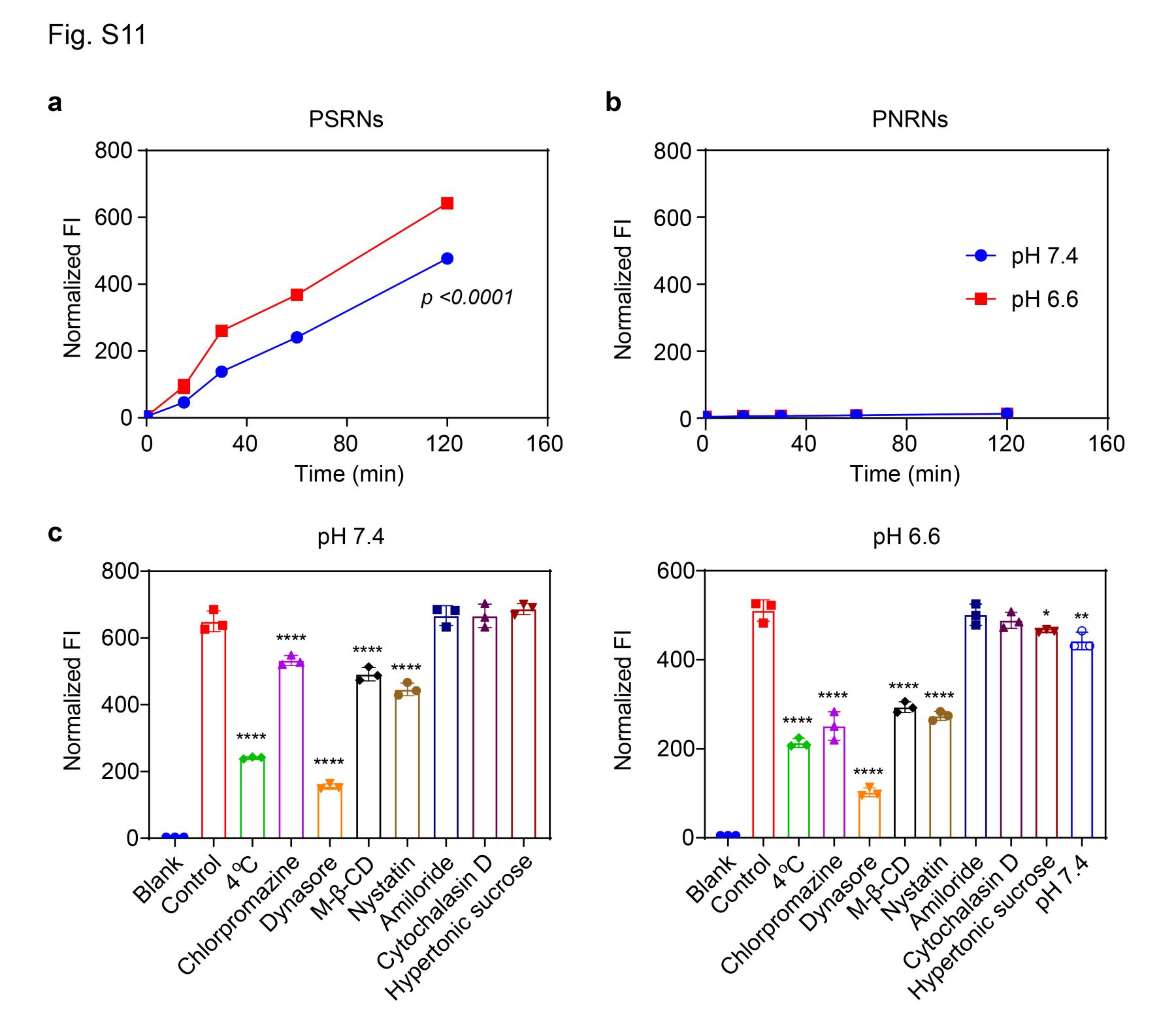


**Figure. S11.** Cell uptake of PSRNs (**a**) and PNRNs (**b**) in CT26 cells at pH 7.4 or 6.6. Mean ± SD (n = 3). **c**. Endocytosis inhibition assay on CT26 cells at pH 7.4 (left) and 6.6 (right). Mean ± SD (n = 3). **p* < 0.05; ***p* < 0.01; *****p* < 0.0001. Statistical analysis in (**a**) was performed by unpaired t-test. Statistical analysis in (**c**) was performed by one-way analysis of variance (ANOVA).

Figure. S12.


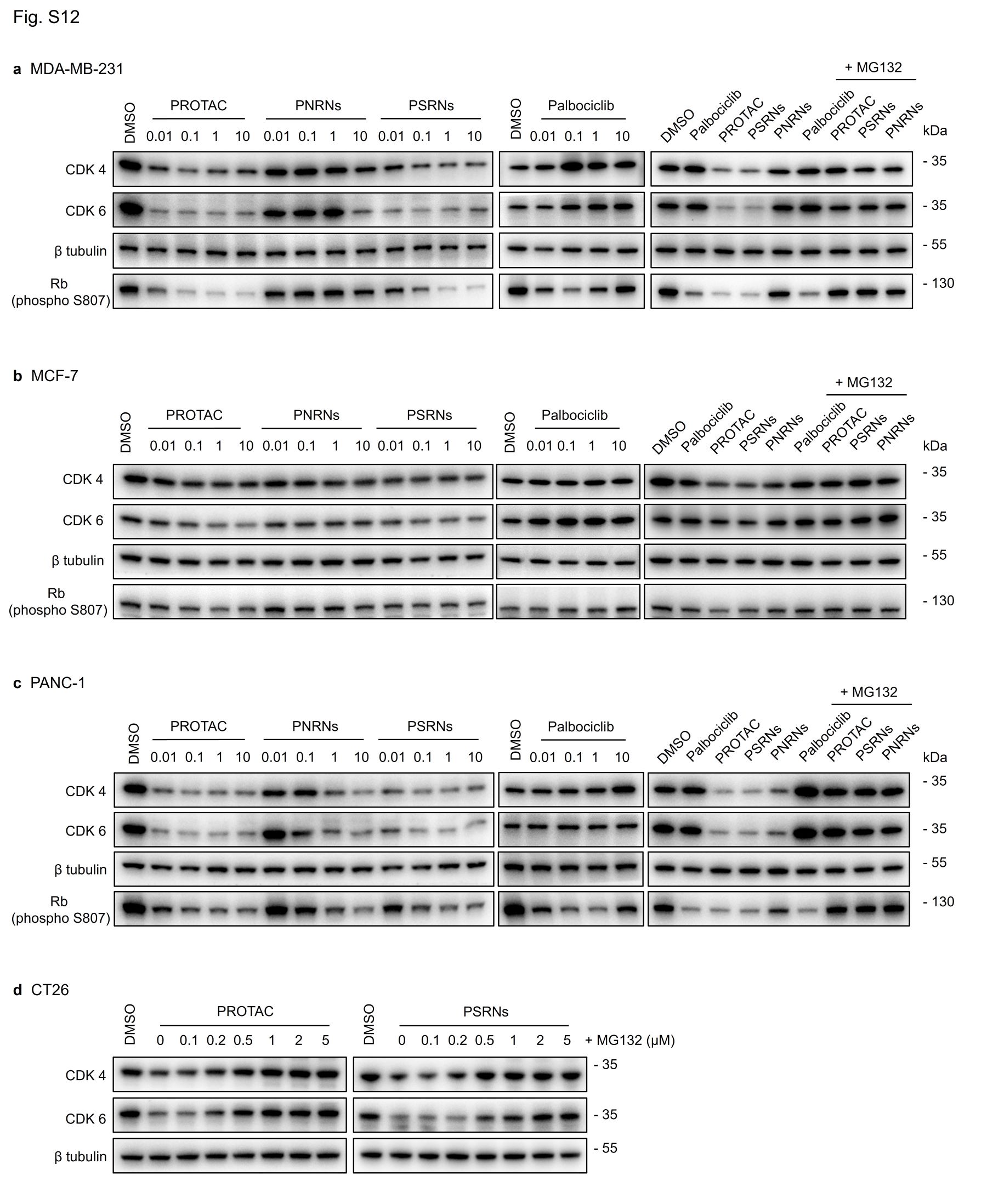


**Figure. S12**. Western blot assay of CDK4/6 and Rb (phospho S807) expression in MDA-MB-231 (**a**), MCF-7 (**b**), PANC-1 (**c**) cells treated with different concentrations (μM) of PROTAC formulations for 16 h. Western blot assay of CDK4/6 and Rb (phospho S807) expression in MDA-MB-231 (**a**), MCF-7 (**b**), PANC-1 (**c**) cells treated w/ or w/o MG132 incubation (PROTAC concentrations of 1.0 μM and MG132 concentration of 0.5 μM). **d**. Western blot assay of CDK4/6 expression in CT26 cells co-treated with different concentrations (μM) of MG132 and 1 μM of PROTAC formulations for 16 h. β-tubulin was used as loading control.

Figure. S13.


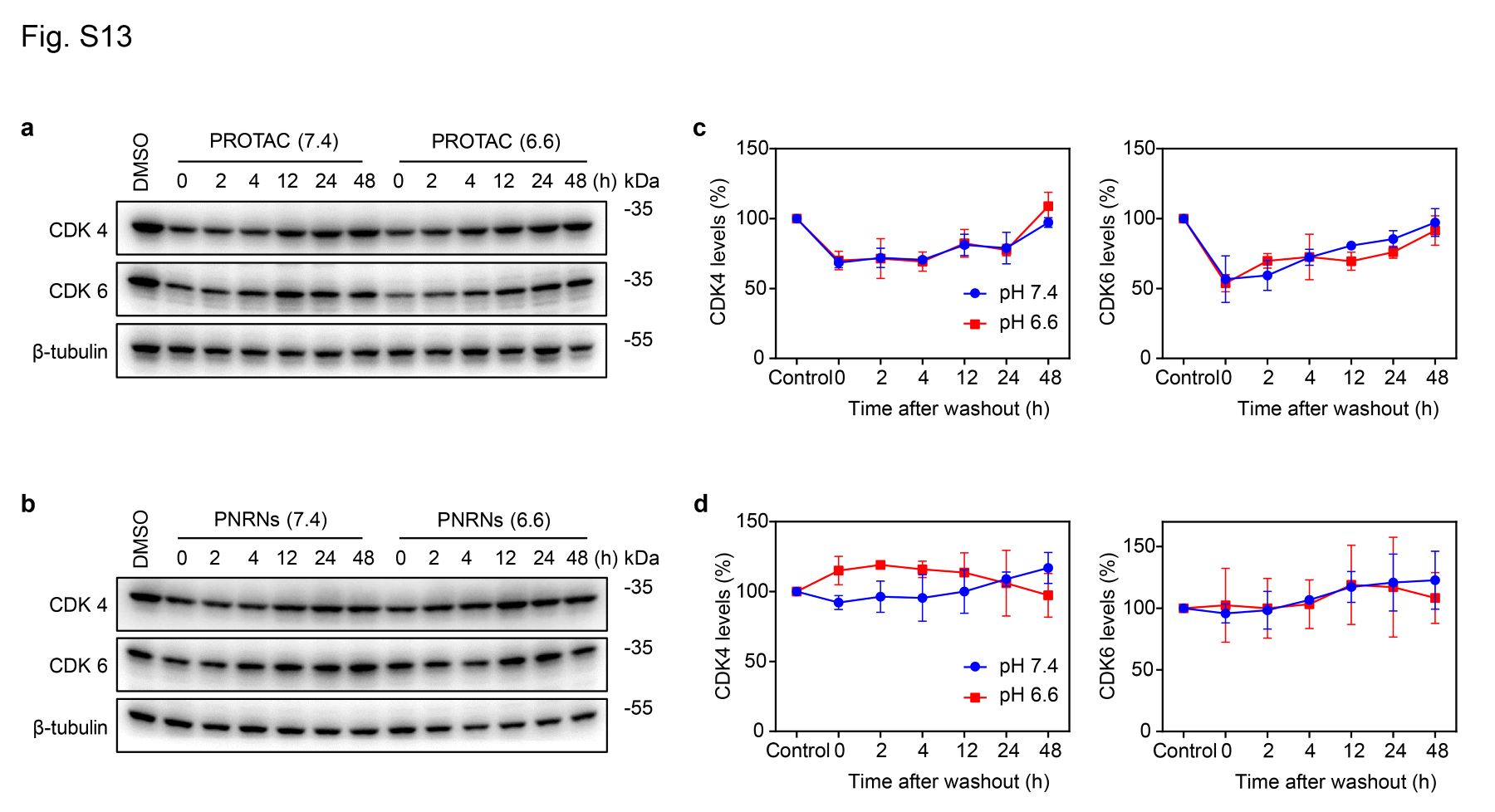


**Figure. S13.** Western blot (**a**, **b**) and quantification (**c**, **d**) of change of CDK4/6 expression in CT26 cells in wash-out assay. Cells were incubated for indicated duration after drug-containing medium replaced by blank medium. Cells were treated with free PROTAC in **a** and **c**, with PNRNs in **b** and **d** (PROTAC concentrations of 1μM). β-tubulin was used as loading control. Mean ± SD (n = 3).

Figure. S14.


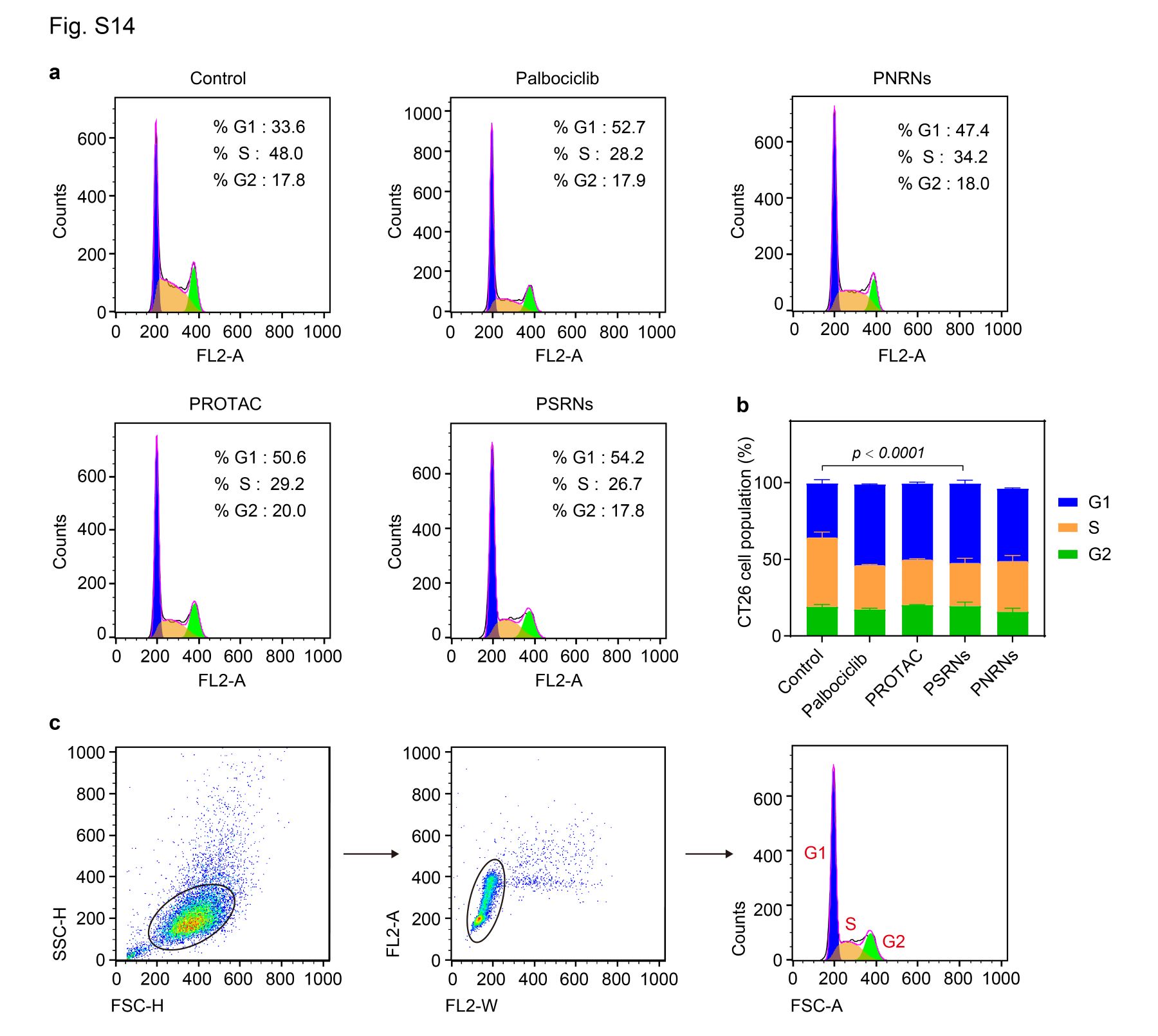


**Figure. S14.** Flow cytometric analysis (**a**) and quantification (**b**) of cell cycle arrest in CT26 cells treated with different PROTAC formulations for 48 h (PROTAC concentration of 1 μM). Mean ± SD, (n = 3). **c**. Gating strategy for cell cycle analysis in (**a**) by flow cytometry. Statistical analysis was performed by one-way analysis of variance (ANOVA).

Figure. S15.


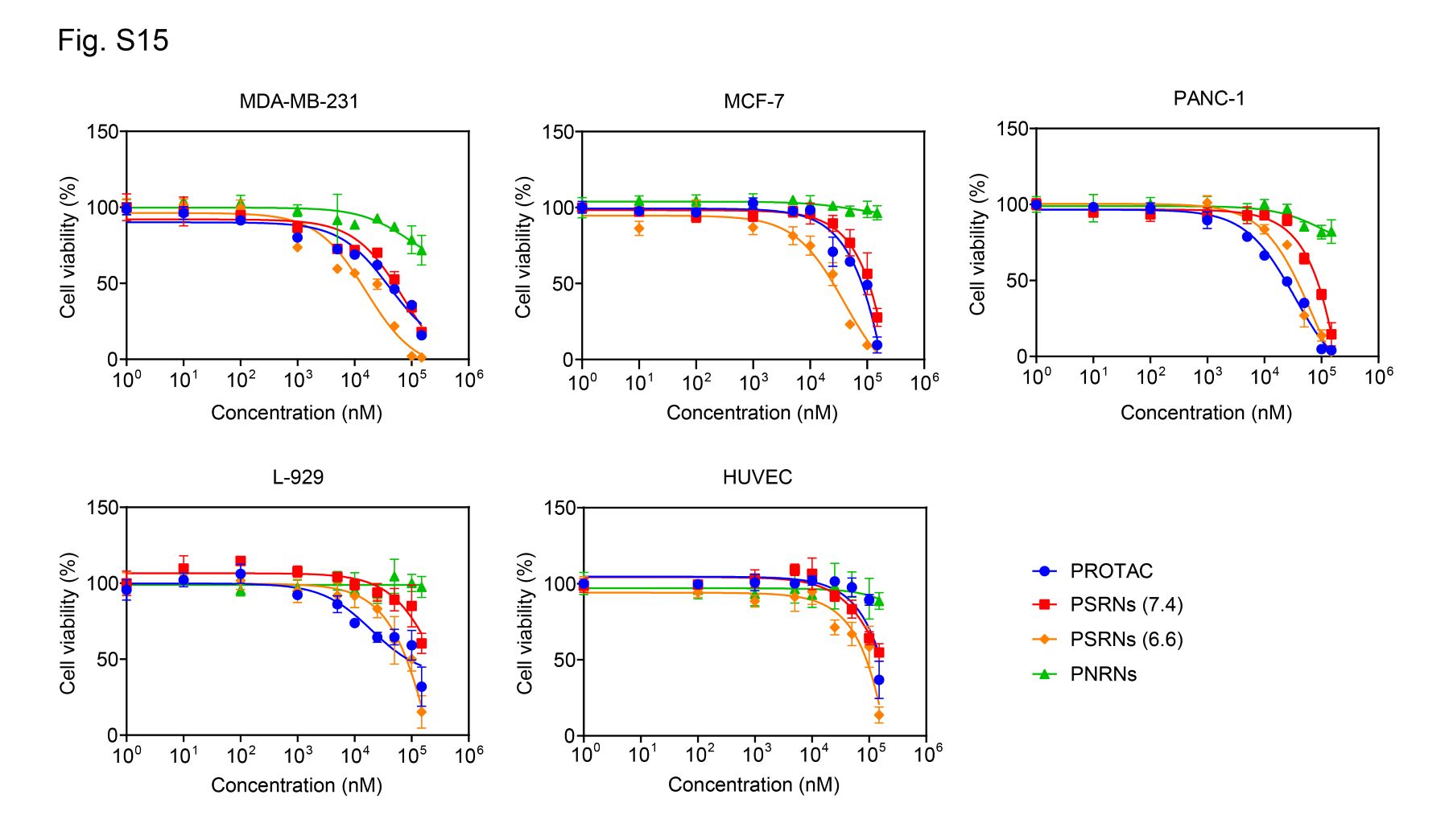


**Figure. S15.** *In vitro* cell viabilities of variety of cells after 72 h incubation with different PROTAC formulations (calculated based on concentration of PROTAC). Mean ± SD, n = 4.

Figure. S16.


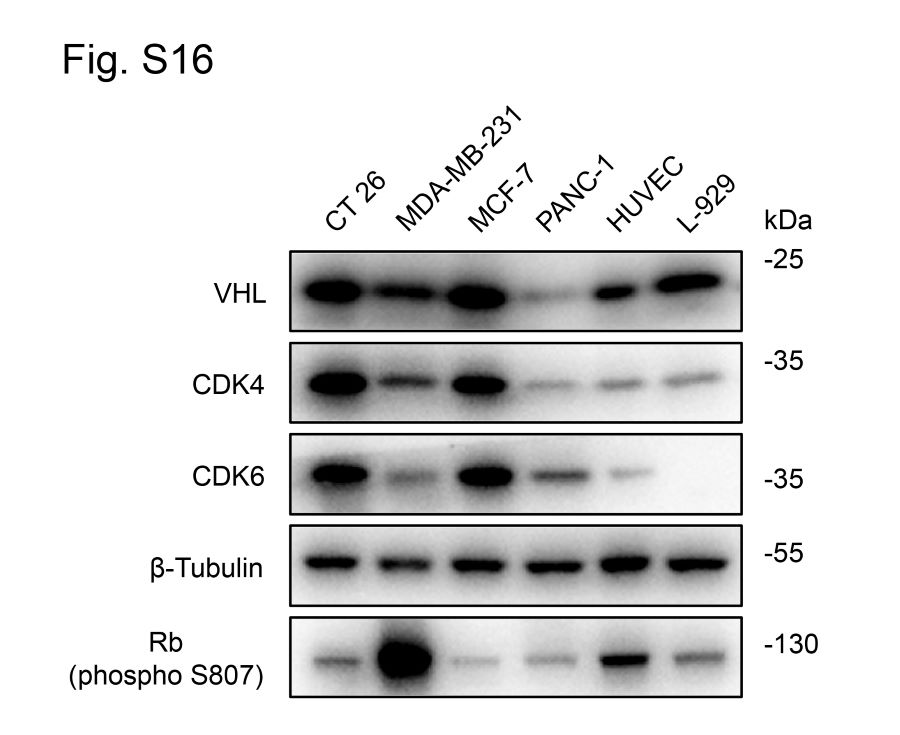


**Figure. S16.** Western blot assay of CDK4/6, VHL and Rb (phospho S807) protein expression in variety of cells. β-tubulin was used as loading control.

Figure. S17.


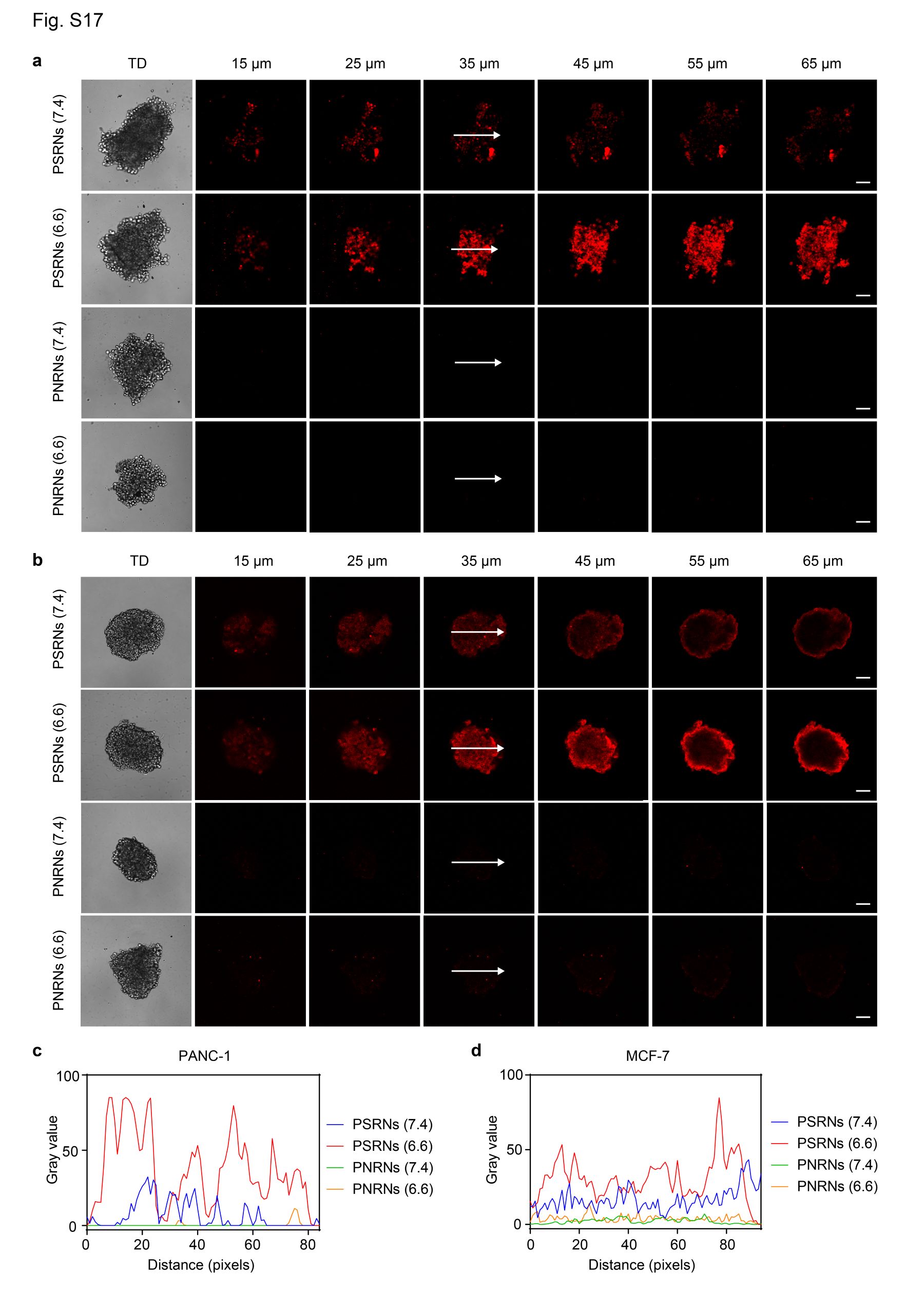


**Figure. S17.** Confocal laser scanning microscopy (CLSM) Z-stack scanning of PANC-1 (low permeability, **a**) and MCF-7 multicellular tumor spheroids (high permeability, **b**) after treatments with PSRNs and PNRNs at pH 7.4 and 6.6 for 8 h. The surface of the spheroids was defined as 0 µm. Scale bar: 100 μm. Cy5 fluorescence intensity along the white lines in PANC-1 (**c**) and MCF-7 (**d**) tumor spheroids’ Z-stack images in (**a**) and (**b**), quantified by ImageJ.

Figure. S18.


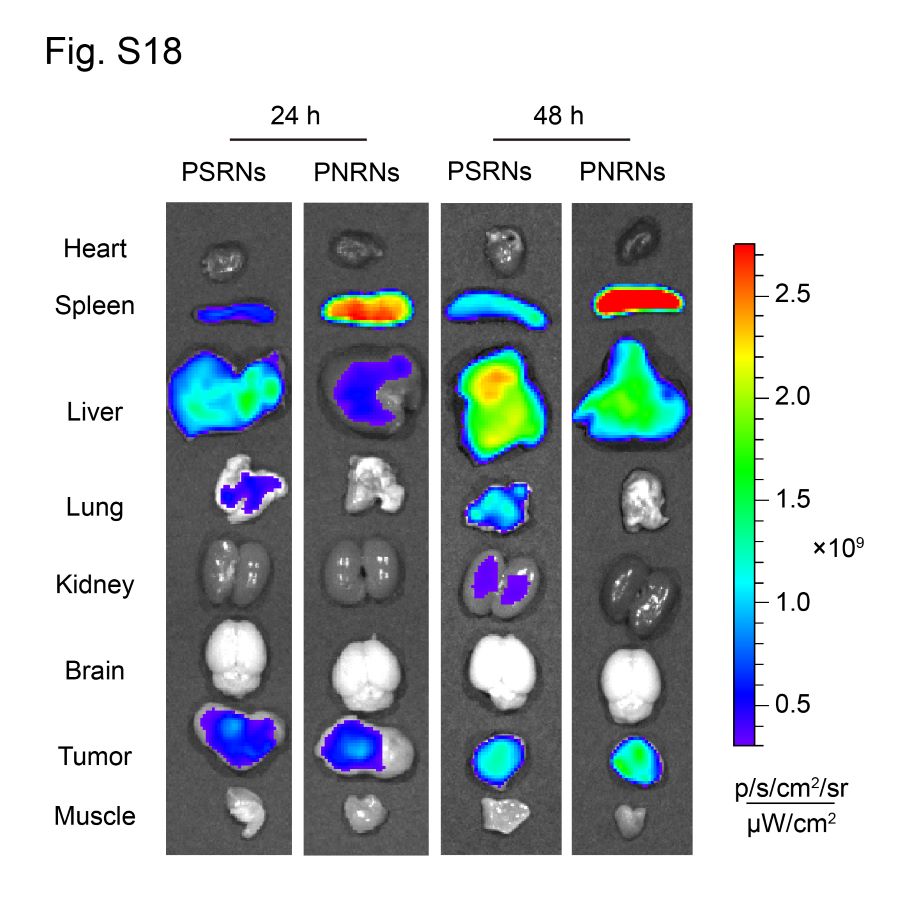


**Figure. S18.** Fluorescence images of the major organs at 24 h and 48 h post-injection of Cy5-labeled preparations.

Figure. S19.


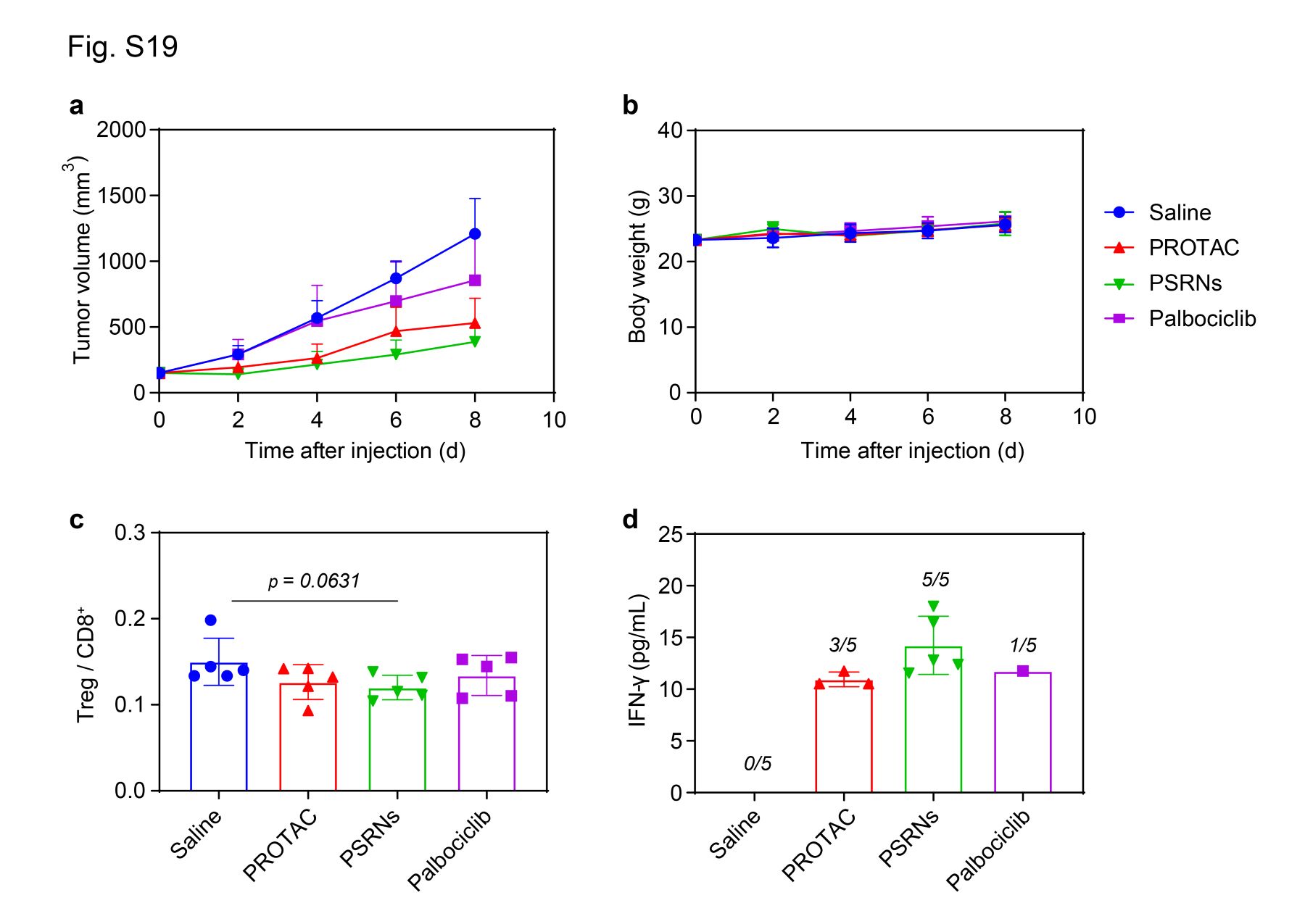


**Figure. S19.** Averaged tumor growth curves (**a**) and body weight changes (**b**) of CT26-tumor bearing BALB/c mice after treatment with different formulations as schedule in Fig. 5**d**. **c**. Treg cells (CD4^+^CD25^+^FoxP3^+^) / CD8^+^ T cells ratios quantified by flow cytometry in lymph nodes. **d**. Levels of IFN-γ in plasma of CT26-tumor bearing BALB/c mice after treatment as schedule in Fig. 5**d** quantified by ELISA assay. Mean ± SD, n = 5. Statistical analysis in (**c**) was performed by unpaired t-test.

Figure. S20.


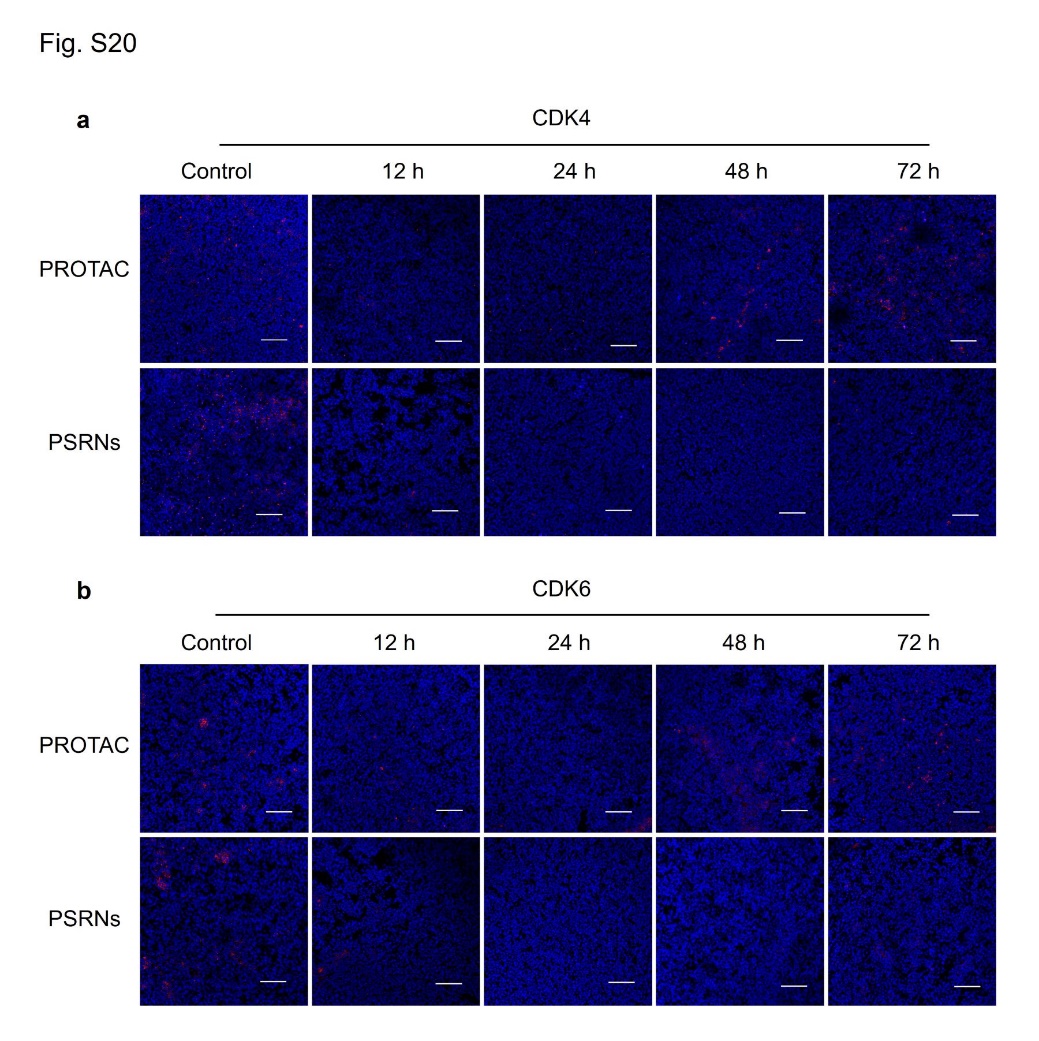


**Figure. S20.** CLSM images of tumor sections for CDK4 (**a**) and CDK6 (**b**) expression after single *i.v.* administration with free PROTAC and PSRNs (PROTAC dosage of 5 mg/kg). Scale bar = 50 μm.

Figure. S21.


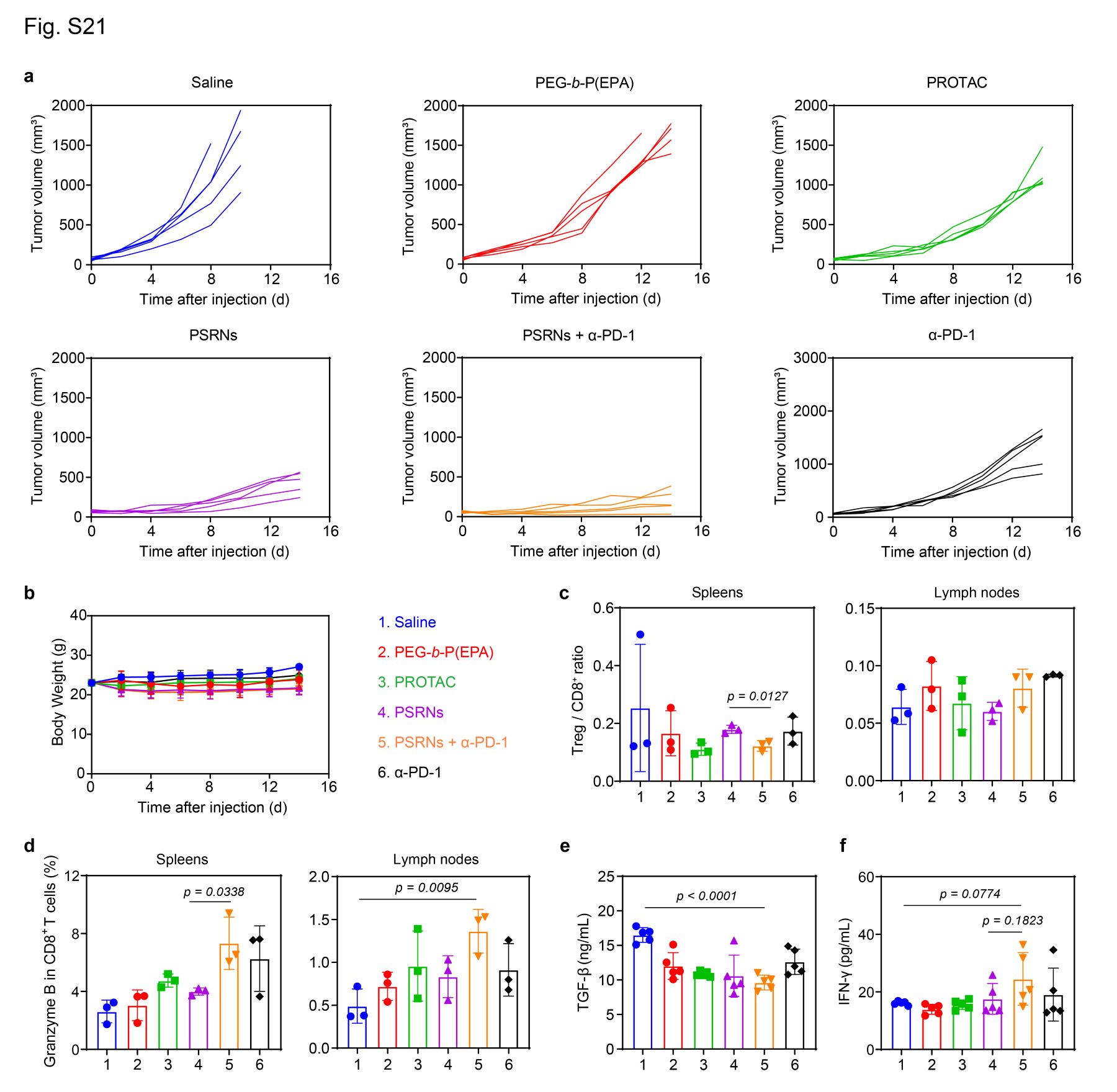


**Figure. S21.** Combination treatment showed the most effective tumor growth inhibition in CT26-tumor bearing BALB/c mice model. Individual tumor growth curves (**a**), body weight changes (**b**) after treatment as experiment schedule in Fig. 6**c**. Mean ± SD, n = 5. Treg cells (CD4^+^CD25^+^FoxP3^+^) / CD8^+^ T cells ratios (**c**) and percents of granzyme B^+^ CD8^+^ T cell populations (**d**) in spleens and lymph glands quantified by flow cytometry. Mean ± SD (n = 3). Levels of TGF-β (**e**) and IFN-γ (**f**) in plasma of CT26-tumor bearing BALB/c mice by ELISA assay. Statistical analysis was performed by unpaired t-test.

Figure. S22.


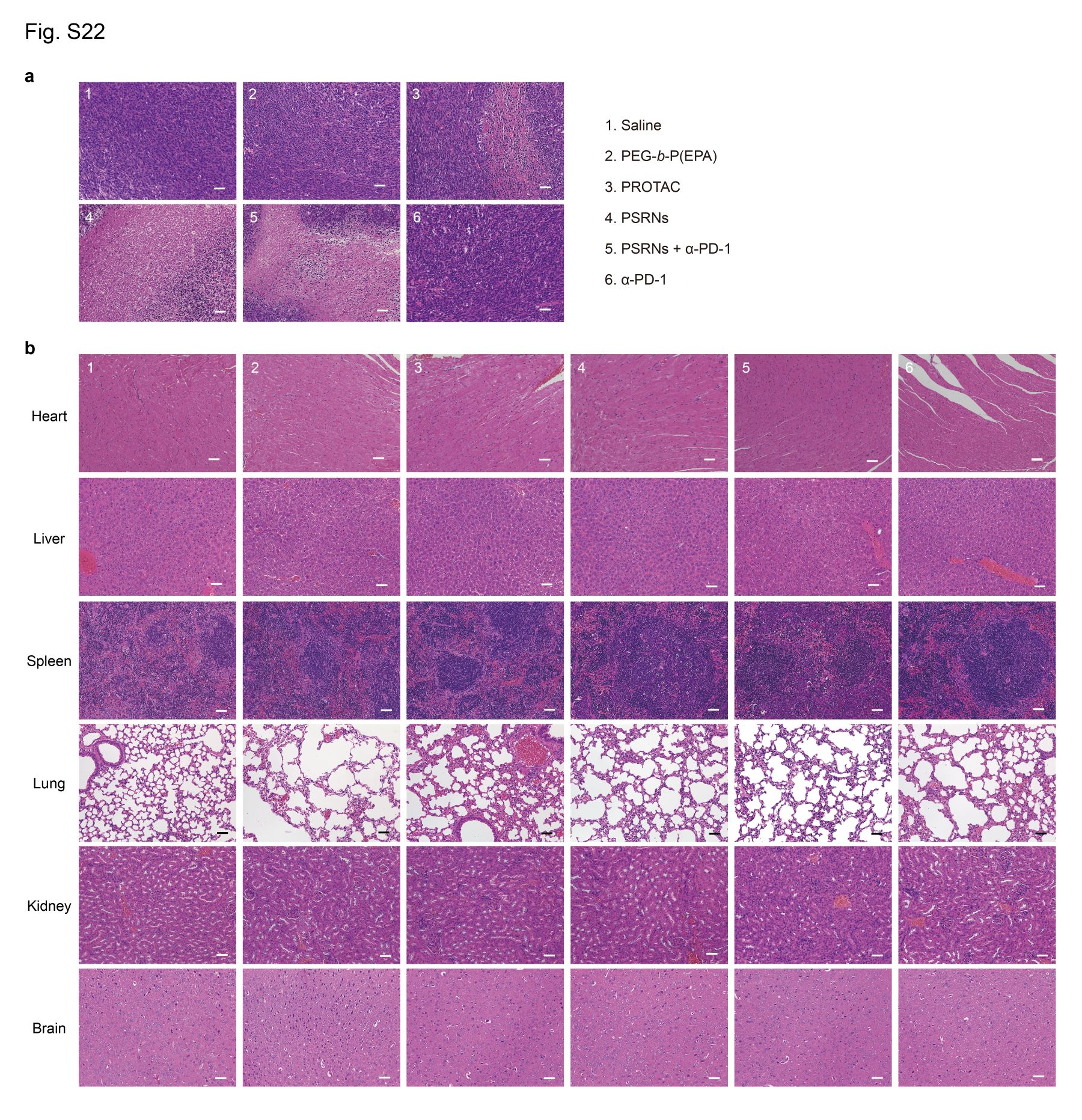


**Figure. S22.** H&E images of CT26 tumor tissues (**a**) and major organs (**b**) harvested from CT26-tumor bearing BALB/c mice treated as Fig. 6**c**. PSRNs combination with immunotherapy showed the most obvious tumor necrosis in CT26 tumor tissues. No obvious tissue damages in mice of different groups were observed. Scale bar = 50 μm.

Figure. S23.


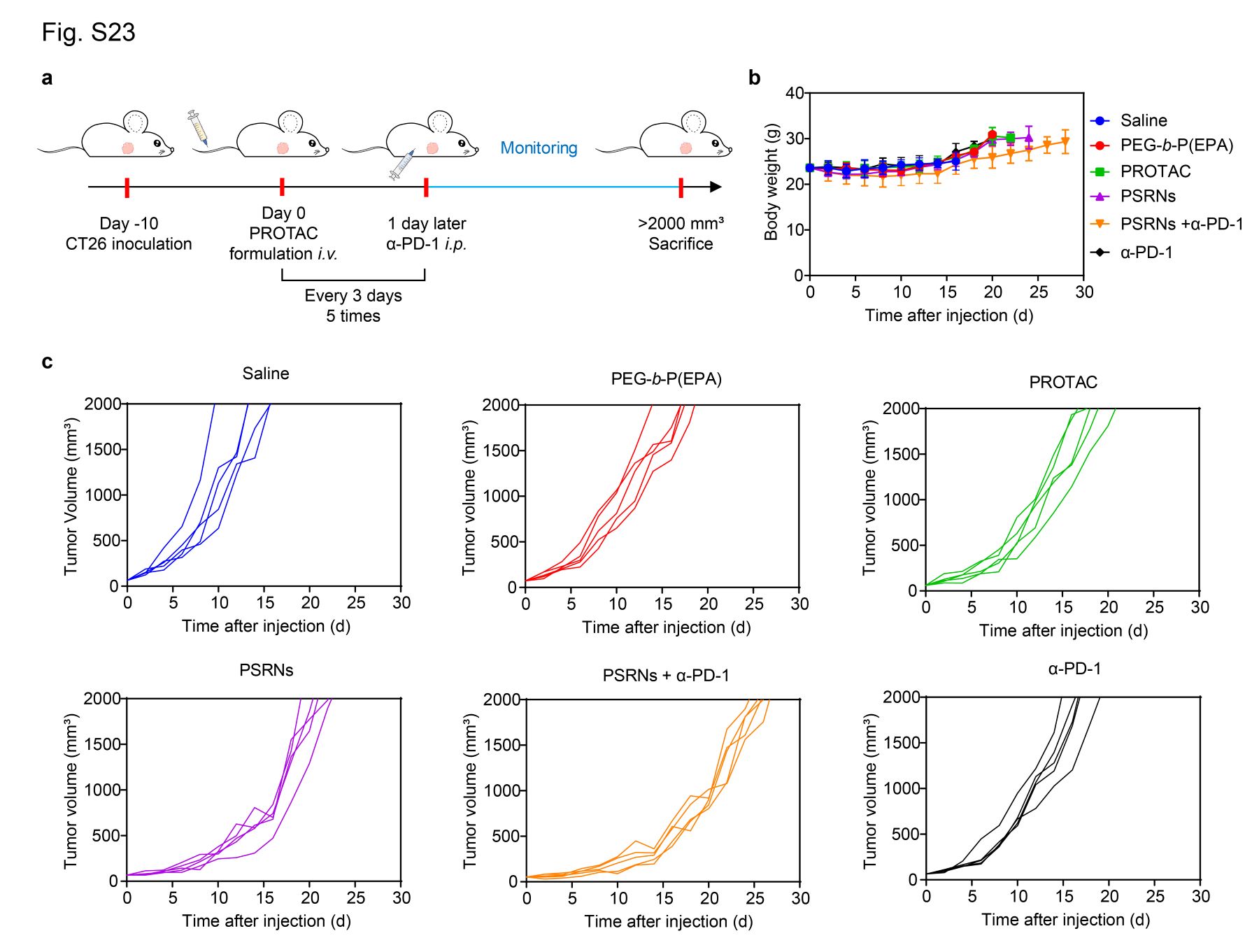


**Figure. S23.** Treatment schedule for survival experiment (related to Fig. 6**e**) in CT26 tumor-bearing BALB/c mice (**a**, n = 5). Body weight changes (**b**) and individual tumor growth curves (**c**) of CT26 bearing BALB/c mice after treatment with different formulations. Mean ± SD, n = 5.

Figure. S24.


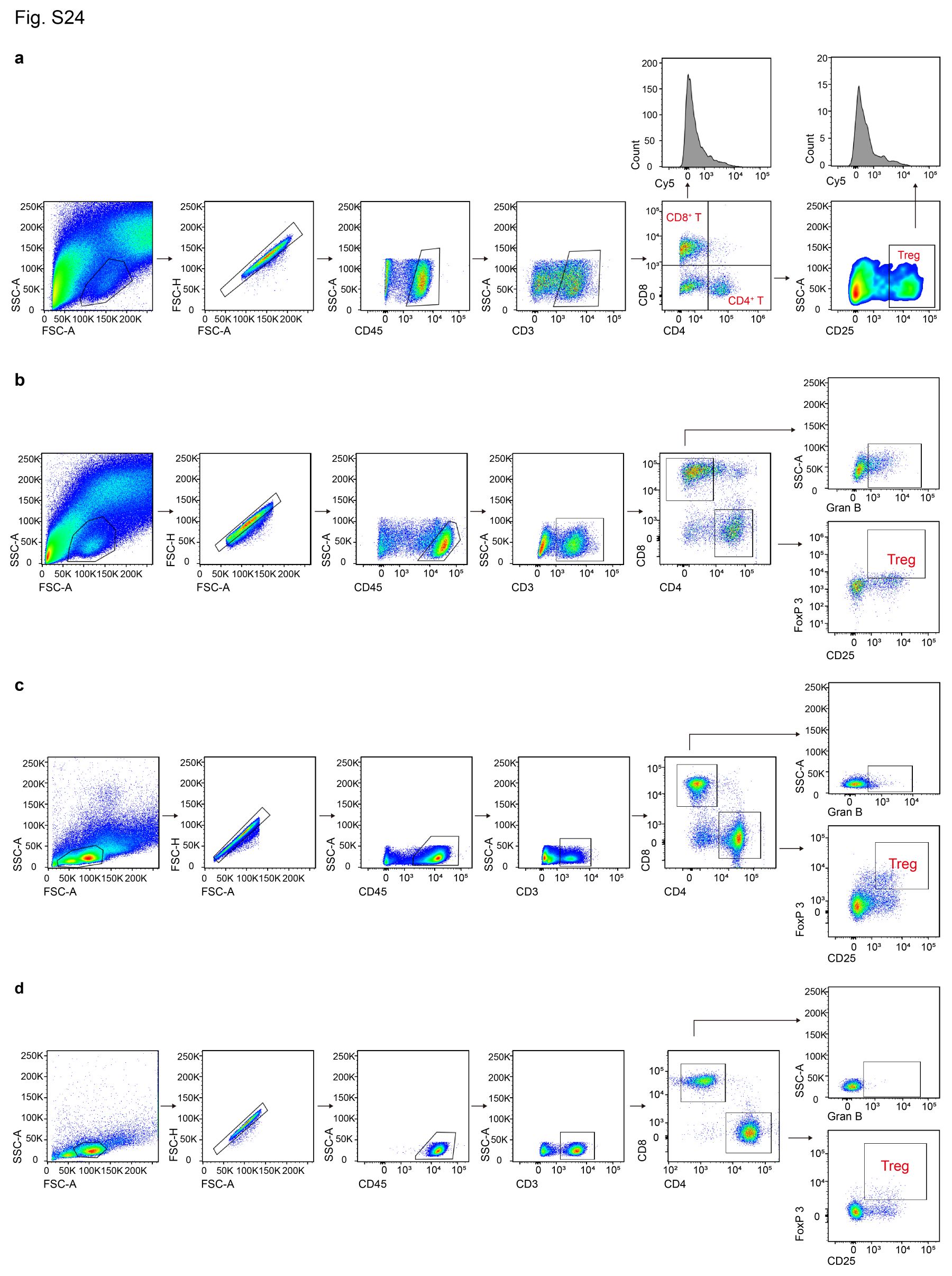


**Figure. S24**. Flow cytometry analysis gating strategies for uptake of PSRNs in Treg cells and CD8^+^ T cells (**a**, related to Fig. 5**g**-**h**), and analysis of immune cells *in vivo* (**b-d**). Gating strategy to analyze Treg cells (CD4^+^CD25^+^Foxp3^+^) and granzyme B-expressed CD8^+^T cells (CD3^+^CD8^+^GranB^+^) from the tumors (**b**, related to Fig. 6**g-h**), spleens (**c**) and tumor-draining lymph nodes (**d**, related to supplementary Fig. S21**c-d**) in CT26 tumor-bearing mice.

Table S1. GPC-determined molecular weights of the diblock copolymers synthesized in this study.

|  | Retention time (min) | M*w* (kDa) | M*n* (kDa) | PDI (M*w*/M*n*) |
| --- | --- | --- | --- | --- |
| PEG-*b*-P(EPA) | 15.3 | 18.6 | 15.9 | 1.17 |
| PEG-*b*-P(EPA-*r*-PROTAC) | 15.1 | 22.7 | 18.2 | 1.25 |
| PEG-*b*-P(EH) | 15.2 | 21.3 | 17.8 | 1.20 |
| PEG-*b*-P(EH-*r*-PROTAC) | 15.0 | 26.5 | 20.0 | 1.33 |

Table S2. CDK4/6 protein degradation percentage (%) after washout with post-incubation of 16 h (PROTAC concentrations of 1μM, groups treated with DMSO were used as 100%). Mean ± SD (PROTAC and PNRNs group, n = 3; PSRNs group, n = 4). **p* < 0.05; ***p* < 0.01.

| pH | PROTAC | | PSRNs | | PNRNs | |
| --- | --- | --- | --- | --- | --- | --- |
|  | CDK4 | CDK6 | CDK4 | CDK6 | CDK4 | CDK6 |
| 7.4 | 17.0±4.7 | 17.2±4.0 | 17.2±8.9 | 16.3±2.5 | -4.1±6.6 | -12.7±12.7 |
| 6.6 | 14.9±2.6 | 22.6±4.6 | 35.9±10.2^*^ | 36.9±6.1^**^ | -7.3±12.7 | -9.9±25.7 |

Table. S3a. IC_50_ values (μM) of CT26 cells in 72 h incubation with different PROTAC formulations.

| pH | PROTAC | PSRNs | PNRNs | SRNs | NRNs |
| --- | --- | --- | --- | --- | --- |
| 7.4 | 21.0 | 21.5 | N.A. | N.A. | N.A. |
| 6.6 | 23.9 | 7.8 | N.A. | 2.3 mg | N.A. |

**Table. S3b**. IC_50_ values (μM) of variety of cells in 72 h incubation with different PROTAC formulations.

|  | pH | MDA-MB-231 | MCF-7 | PANC-1 | L-929 | HUVEC |
| --- | --- | --- | --- | --- | --- | --- |
| PROTAC | 7.4 | 35.8 | 79.9 | 22.8 | N.A. | 85.6 |
| PSRNs | 7.4 | 48.9 | 106.1 | 83.6 | N.A. | N.A. |
|  | 6.6 | 12.8 | 25.7 | 36.3 | 90.0 | 81.6 |
| PNRNs | 7.4 | N.A. | N.A. | N.A. | N.A. | N.A. |

Table. S4. Pharmacokinetic parameters of PSRNs and PNRNs in health balb/c mice. (n=5)

|  | t_1/2β_ (h) | AUC_(0-∞)_ (mg/L*h) | MRT_(0-∞)_ (h) |
| --- | --- | --- | --- |
| PSRNs | 12.548±7.179 | 747.876±64.505 | 53.127±12.556 |
| PNRNs | 13.668±1.014 | 1383.017±163.684 | 22.63±2.587 |

Table. S5. *In vivo* CDK4/6 protein degradation percentage (%) after single administration (PROTAC dosage of 5mg/kg, *i.v.*). Tumors harvested from untreated mice were used as 100%. Mean ± SD (n = 3). **p* < 0.05; ***p* < 0.01.

|  | PROTAC (sol.) | PSRNs |
| --- | --- | --- |
| CDK4 | 13.8±5.7 | 36.1±9.2^**^ |
| CDK6 | 18.4±4.8 | 54.5±16.1^*^ |

Table. S6. Median survival times (MSTs) after treatment with different formulations (related to Fig. 6e).

|  | Saline | PEG-*b*-P(EPA) | PROTAC | PSRNs | PSRNs + α PD-1 | α PD-1 |
| --- | --- | --- | --- | --- | --- | --- |
| MST (day) | 14 | 18 | 20 | 22 | 26 | 18 |
