# Supplementary material for: Sequential responsive nano-PROTACs for precise intracellular delivery and enhanced degradation efficacy in colorectal cancer therapy
Source: Signal Transduct Target Ther. 2024 Oct 18;9:275. doi: 10.1038/s41392-024-01983-1 (PMC11486899; doi:10.1038/s41392-024-01983-1)
Supplement: Supplementary file 3 — Raw data of western blot assays [file 41392_2024_1983_MOESM3_ESM.docx]

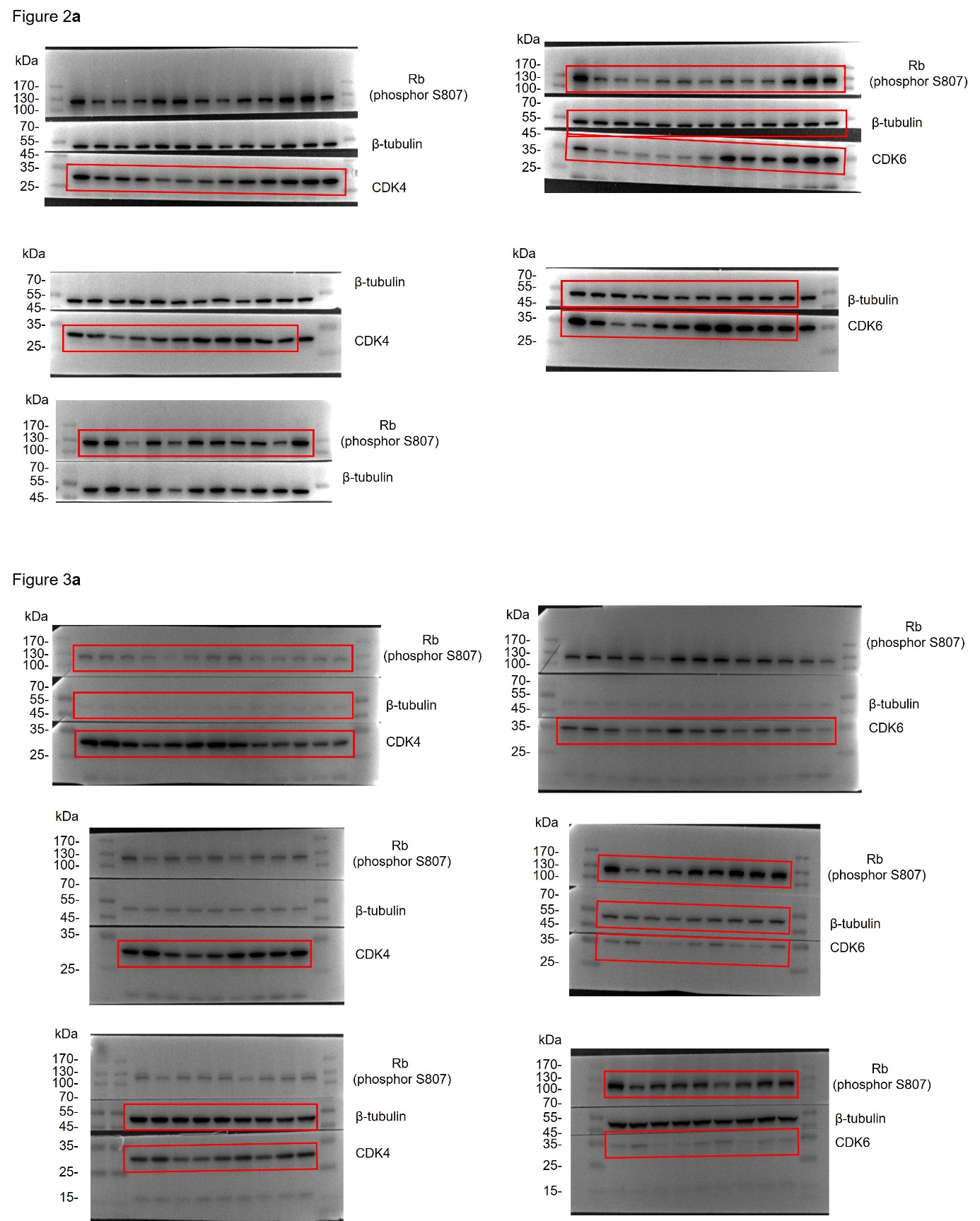


Raw data of western blot assays in Figure 2**a** and 3**a**.


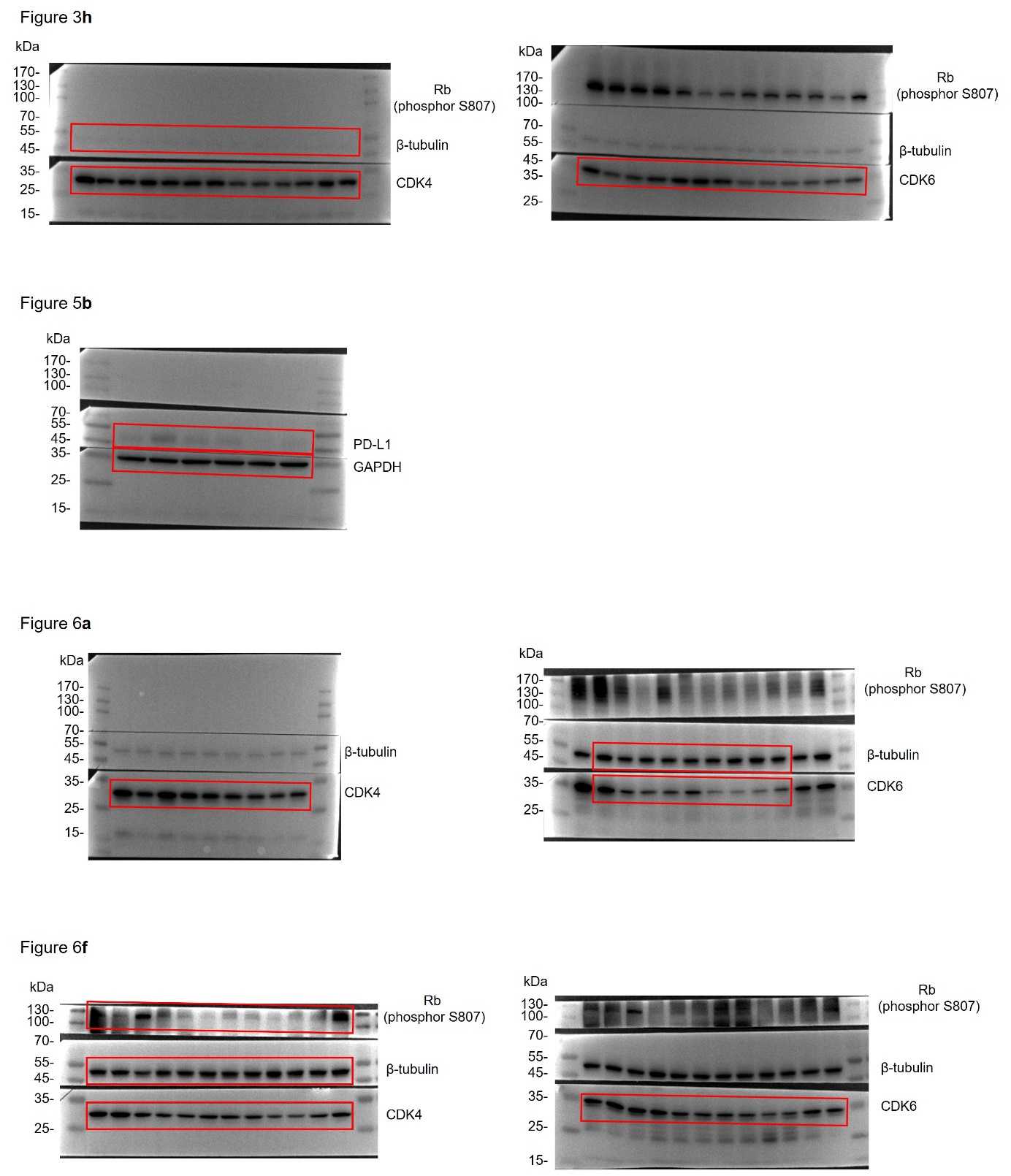


Raw data of western blot assays in Figure 3**h**, 5**b**, 6**a** and 6**f**.


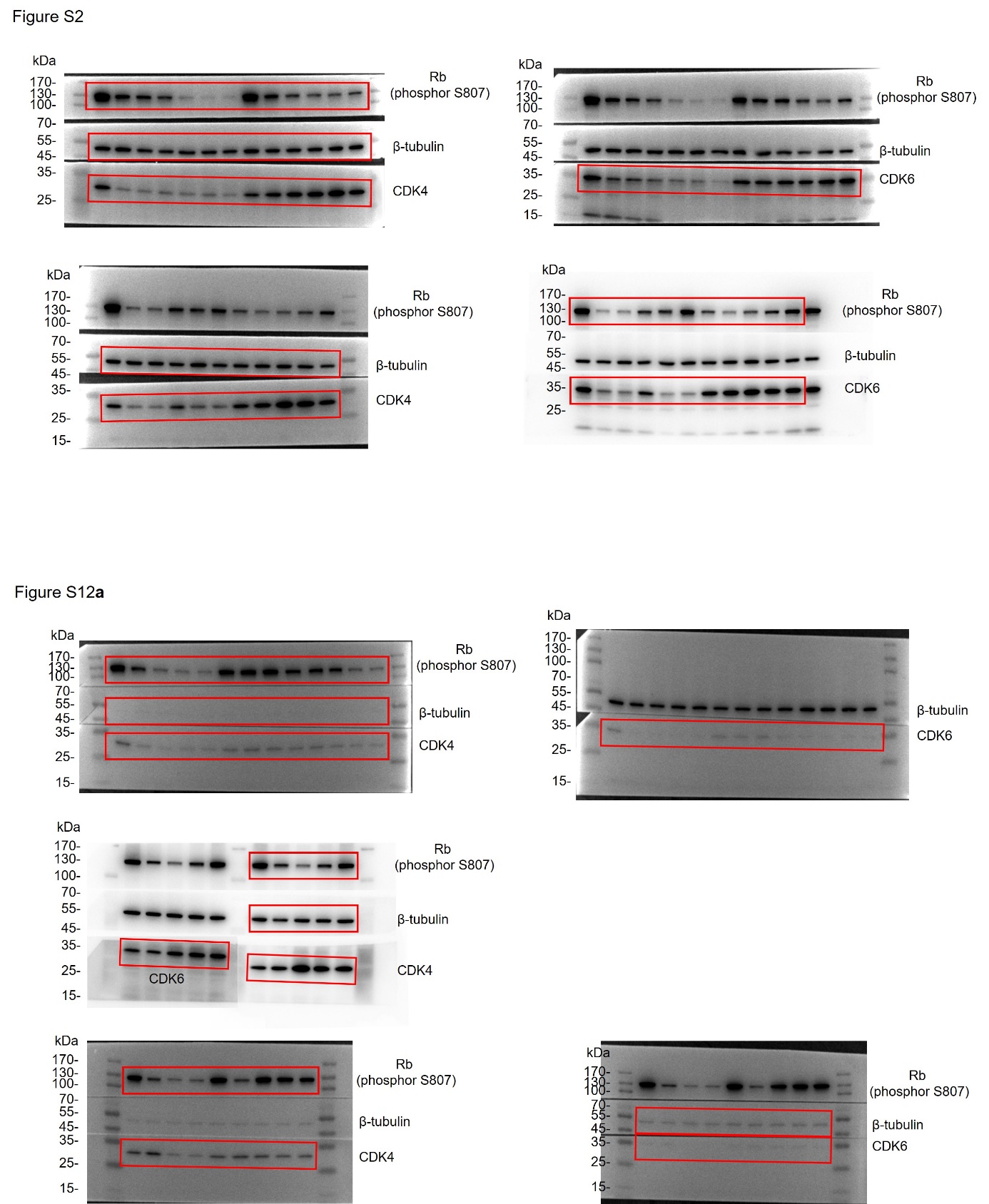


Raw data of western blot assays in Figure S2 and S12**a**.


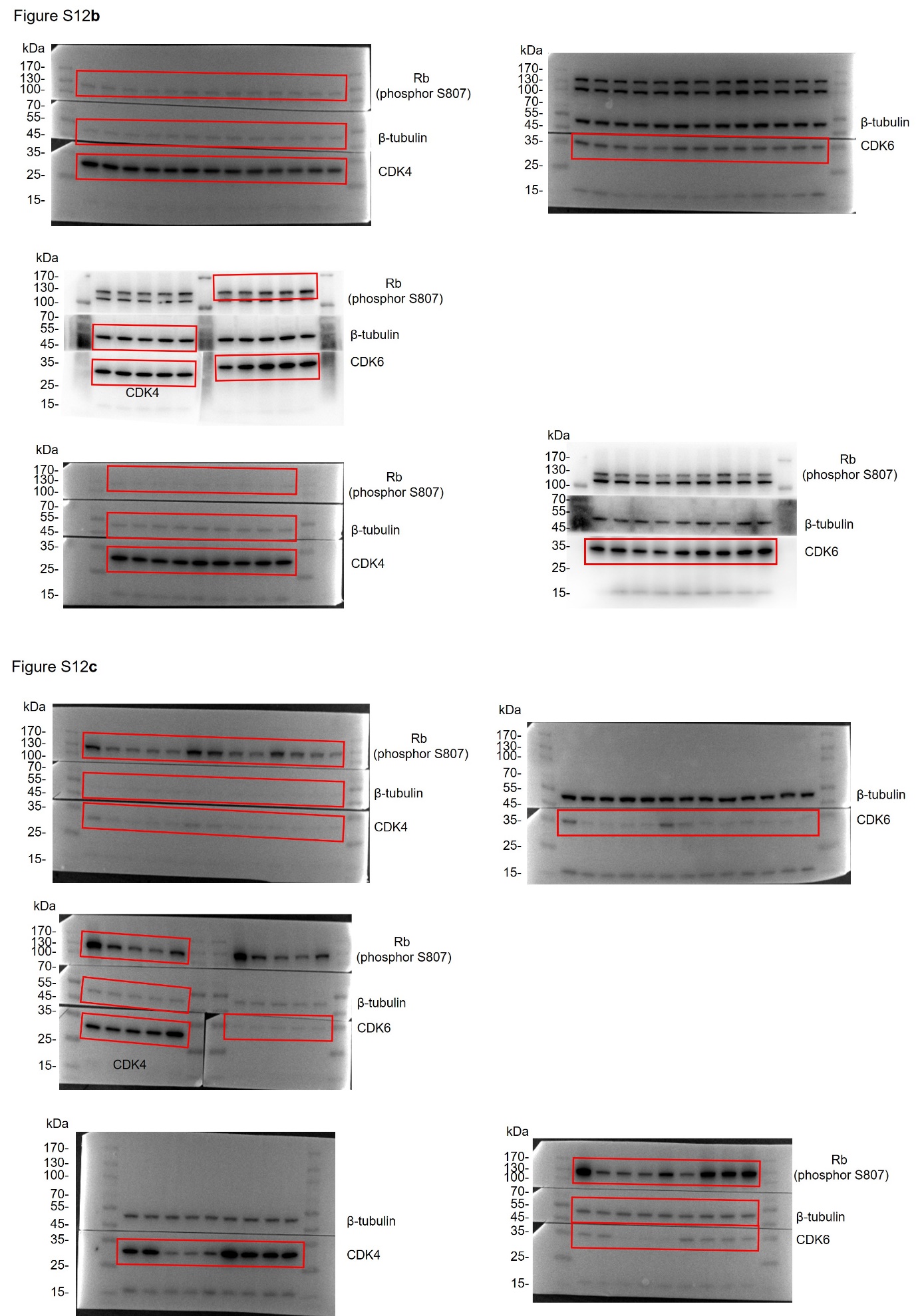


Raw data of western blot assays in Figure S12**b** and S12**c**.


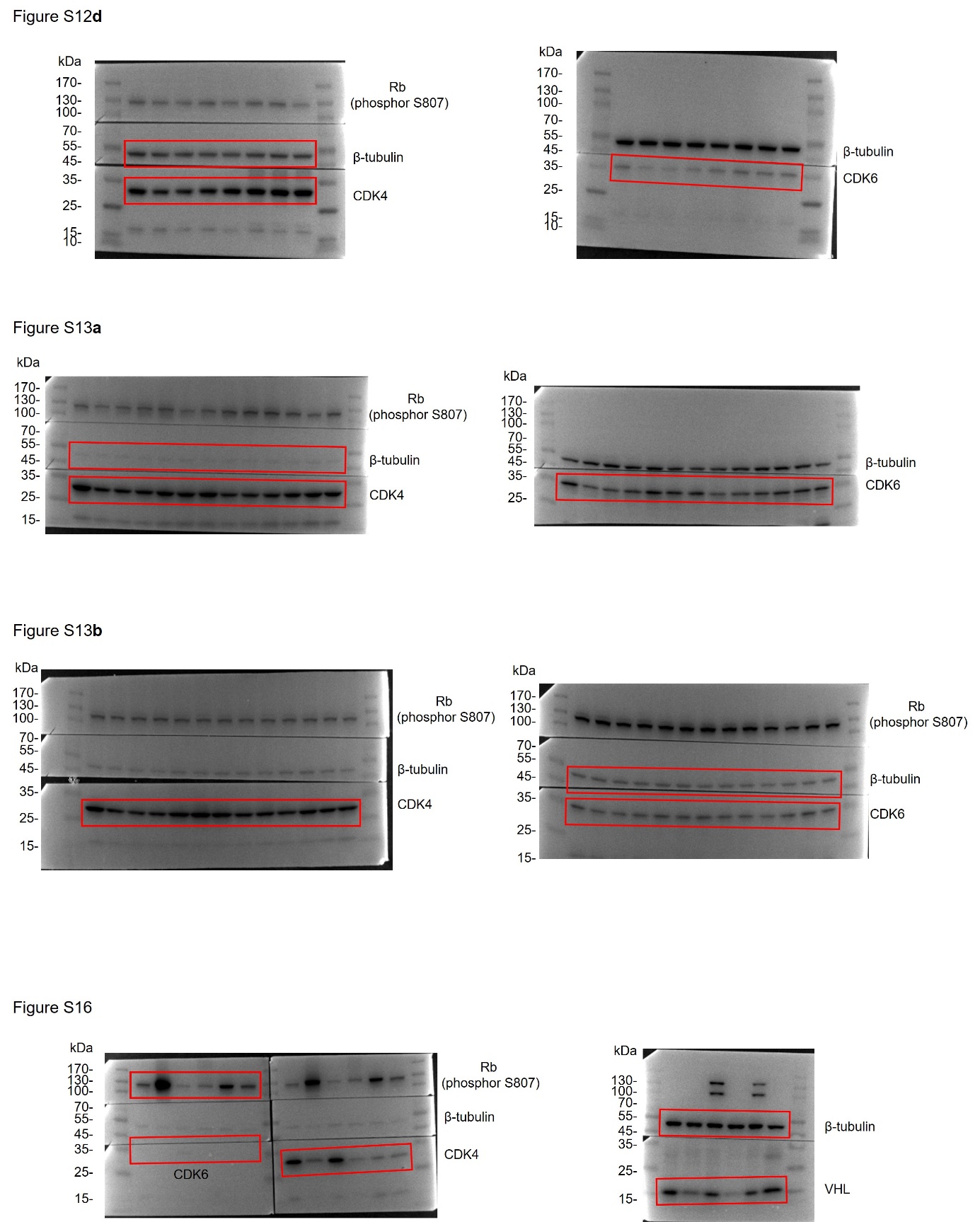


Raw data of western blot assays in Figure S12**d**, S13**a**, S13**b** and S16.
